# Multimodal Evaluation of [^225^Ac]Ac-PSMA-617 Therapy in mCRPC: PET Based Total Tumor Volume, PSA Kinetics, and Blood Biomarkers

**DOI:** 10.3390/ijms27209330

**Published:** 2026-10-20

**Authors:** Ali Ebrahimifard, Shamim Bagheri, Qi Wang, Azimeh Hojjat Shamami, Pietro Di Fazio, Friederike Eilsberger, Laura Müller, Markus Luster, Damiano Librizzi, Behrooz Hooshyar Yousefi

**Affiliations:** 1 Department of Nuclear Medicine, School of Medicine, Philipps-University Marburg, 35043 Marburg, Germany; 2 Department of Urology, School of Medicine, Philipps-University Marburg, 35043 Marburg, Germany; 3 Department of Nuclear Medicine, University Hospital OWL, Evangelical Hospital Bethel, 33617 Bielefeld, Germany

**Keywords:** metastatic castration-resistant prostate cancer (mCRPC), prostate-specific antigen (PSA), targeted α-therapy (TAT), [^225^Ac]Ac-PSMA-617, [^177^Lu]Lu-PSMA, total tumor volume (TTV), PSA kinetics, blood biomarkers, salivary glands uptake, therapeutic efficacy, overall survival (OS), Median progression-free survival (PFS), biomarker-guided evaluation

## Abstract

This retrospective analysis interrogates the value of a multimodal biomarker paradigm to characterize molecular and clinical responses to [^225^Ac]Ac-PSMA-617-targeted α-therapy (TAT) in cohorts of heavily pretreated metastatic castration-resistant prostate cancer patients who had shown limited benefit from prior [^177^Lu]Lu-PSMA-617. By integrating quantitative PSMA-PET/CT metrics—notably total tumor volume (TTV) and lesion SUV indices—with longitudinal PSA kinetics, routine hematologic and biochemical markers, and semiquantitative assessment of salivary gland uptake, we sought to delineate treatment effect, organ-specific off-target impact, and prognostic biomarker signatures. In twelve patients receiving 2–6 cycles of therapy (median activity 8 MBq), biochemical responses were frequent: 8/12 experienced PSA declines and 5/12 achieved ≥50% reduction. Concordant molecular responses were observed on PSMA-PET, with SUV_peak_-based partial response in 7/12, stable disease in 3/12, and progressive disease in 2/12 of the target lesions. Qualitative concordance between TTV and PSA response was observed in four illustrative patients; no formal cohort-level concordance analysis was performed. Notably, salivary gland tracer uptake on [^68^Ga]Ga-PSMA-11 PET/CT decreased after two cycles of [^225^Ac]Ac-PSMA-617 compared with baseline (SUV_max_ −63.7%; SUV_mean_ −45.5%). In this exploratory cohort, elevations in PSA, alkaline phosphatase, and LDH measured after two cycles of therapy, along with low hemoglobin, platelet count, and creatinine, were associated with shorter overall survival. Median progression-free survival and overall survival were 27 and 53 weeks, respectively. Collectively, these findings suggest that a combined PSMA-PET TTV–PSA–blood biomarker framework may offer complementary insight alongside PSA alone for characterizing therapeutic response, although its incremental value over PSA was not formally tested in this exploratory cohort; blood-based biomarkers (PSA, ALP) showed the most consistent prognostic association with survival, providing objective measures of both tumor response and collateral tissue impact. Prospective validation in larger, controlled cohorts is warranted to refine biomarker thresholds and guide personalized TAT strategies.

## 1. Introduction

Metastatic castration-resistant prostate cancer (mCRPC) presents a significant therapeutic challenge, as it usually shows limited or no durable response to newer treatments such as novel antiandrogen agents, taxane-based chemotherapy, or Radium-223 (^223^Ra) [1,2]. In this context, radioligand therapy (RLT) has emerged as a promising alternative, with clinical studies such as the VISION trial demonstrating favorable outcomes for [^177^Lu]Lu-PSMA-617, despite its associated resistance or recurrence rates [3,4,5,6]. These challenges have led researchers to explore the potential of alpha-emitting radionuclides, which offer higher energy transfer rates and shorter path lengths than beta-emitting agents, making them an attractive option for PSMA (Prostate-Specific Membrane Antigen)-targeted RLT in mCRPC [7].

Actinium-225 (^225^Ac), with an alpha particle energy range of 5.8–8.4 MeV and a half-life of 9.9 days, has been widely used in TAT [8,9]. Moreover, ^225^Ac, with multiple alpha-emitting decay products, offer an effective TAT for cancer cells [10]. Since 2017, numerous reports have confirmed that [^225^Ac]Ac-PSMA-617 therapy induces significant declines in prostate-specific antigen (PSA) levels following treatment [11,12,13,14,15,16,17]. Furthermore, it is associated with improved progression-free survival (PFS), especially in patients who have previously been refractory to [^177^Lu]Lu-PSMA-617. Given the promising yet varied clinical outcomes, a comprehensive clinical analysis is essential to provide a holistic understanding of treatment efficacy, patient outcomes, and associated toxicities. This analysis will offer valuable insights into the therapeutic benefit of [^225^Ac]Ac-PSMA-617.

In [^225^Ac]Ac-PSMA-617 therapy, the salivary gland is one of the organs at risk (OAR) due to physiological PSMA expression, leading to potential off-target uptake and xerostomia [18]. Quantifying this uptake is crucial for assessing toxicity, optimizing therapy, and evaluating the tumor sink effect, which occurs when tumor reduction alters biodistribution, increasing uptake in non-target tissues. Semi-quantitative PET analysis represents a pragmatic and essential approach for evaluating treatment outcomes and assessing therapeutic efficacy.

Despite encouraging preliminary clinical results, the relationship between multimodal biomarkers, including blood-based biomarkers and PSMA PET-derived total tumor volume (TTV), and therapeutic efficacy or overall survival (OS) remains insufficiently characterized. Therefore, this retrospective study comprehensively assessed the value of PSA kinetics, PSMA PET-derived TTV and standardized uptake value (SUV) metrics, together with routine blood biomarkers, for monitoring molecular response to [^225^Ac]Ac-PSMA-617 therapy in patients with mCRPC. In addition, we explored the prognostic significance of baseline and post-treatment blood biomarker profiles for OS, addressing an important knowledge gap in the current literature.

To contextualize and compare our findings, we conducted a pooled quantitative analysis to consolidate existing data on the efficacy and safety of [^225^Ac]Ac-PSMA-617 therapy in mCRPC. By integrating outcomes from multiple cohorts, the pooled quantitative analysis provides a descriptive overview of PSA response rates, progression-free survival (PFS), OS, and treatment-related toxicities, including xerostomia, renal dysfunction, and hematologic effects. Through the synthesis of both retrospective findings and pooled evidence, this study provides a robust, evidence-based perspective on therapeutic efficacy, safety, and prognostic biomarkers informing future strategies for treatment optimization.

In this study we adopt a biomarker-centric framework in which quantitative PSMA-PET metrics (TTV and lesion SUV indices), longitudinal PSA kinetics, and routine hematologic and biochemical markers constitute the primary translational endpoints. Clinical endpoints (PFS and OS) are explicitly treated as validation measures to contextualize and support biomarker signals rather than as the sole determinants of efficacy. We further performed focused exploratory prognostic analyses, including bootstrapped confidence intervals and internal validation of candidate prognostic biomarkers to generate hypothesis-forming estimates of prognostic performance. This multimodal framework is intended to complement, rather than to demonstrate superiority over, PSA-based response assessment; whether it offers incremental value beyond PSA alone is addressed as an exploratory question in this analysis (see Discussion). Finally, we interrogated mechanistic links between observed biomarker dynamics and alpha-particle radiobiology, including the tumor sink effect, to provide a biologically coherent interpretation of our findings.

## 2. Results

### 2.1. Patients and Treatment Administration

A total of 12 patients with metastatic castration-resistant prostate cancer were selected who underwent [^225^Ac]Ac-PSMA-617 targeted alpha therapy (Table 1). The patients had received various prior treatments including local treatments, androgen receptor–targeted therapy (AR-TT), chemotherapy, and [^223^Ra]RaCl_2_ before initiation of [^225^Ac]Ac-PSMA-617 TAT. Local treatments included radical prostatectomy (67%), and radiotherapy (42%), both of which remain key in local/locally advanced disease management. Most patients had undergone multiple lines of AR-TT, including abiraterone (75%) and enzalutamide (83%). Recent analyses suggest that AR-TT inhibitors continue to contribute significantly to survival in mCRPC even in patients heavily pretreated by other modalities [19]. Chemotherapy was also widely used: docetaxel (67%), established as first-line chemotherapy in mCRPC [20,21], and cabazitaxel (42%), which showed efficacy after docetaxel failure [22,23]. Notably, one patient (8%) initially referred for [^223^Ra]RaCl_2_ therapy due to extensive osseous metastases demonstrated no response to the [^223^Ra]RaCl_2_ therapy. All patients received [^177^Lu]Lu-PSMA-617 therapy following multidisciplinary Tumor Board approval, ensuring uniform eligibility criteria and therapeutic intent majorly including low/no response or disease progression after prior therapies across the cohort, with a median of 4 cycles (range 2–8). Patients were considered for treatment with [^225^Ac]Ac-PSMA-617 TAT if they showed disease progression, as confirmed by [^68^Ga]Ga-PSMA-11 PET/CT imaging and PSA levels. The distribution of [^225^Ac]Ac-PSMA-617 treatment cycles was as follows: three patients (25%) underwent five cycles, three patients (25%) received three cycles, and another three patients (25%) received two cycles. Additionally, two patients (17%) completed four cycles, and one patient (8%) received six cycles (Figure S1). The median injected radioactivity (injected activity (IA)) was 8 MBq, ranging from 5.0 MBq to 11.0 MBq (approximately IA = 100 kBq/kg).

Baseline serum biomarkers and hematologic parameters were assessed in mCRPC patients prior to administration of [^177^Lu]Lu-PSMA-617 and [^225^Ac]Ac-PSMA-617 therapy. Table 2 summarizes an overview of laboratory values before treatment initiation.

Treatment cycles for each patient are illustrated using a swimmer plot (Figure 1), highlighting the number of [^177^Lu]Lu-PSMA-617 and [^225^Ac]Ac-PSMA-617 administrations as well as intervals between therapies. Patients received 2 to 8 cycles of [^177^Lu]Lu-PSMA-617 and 2 to 6 cycles of [^225^Ac]Ac-PSMA-617.

### 2.2. Safety

No patient exhibited signs or symptoms of urinary tract obstruction, as confirmed by repetition or sequential pre-treatment dynamic renal scintigraphy using [^99m^Tc]Tc-MAG3.

Hematologic and renal biomarkers, including hemoglobin, white blood cell (WBC) count, platelet count, creatinine, estimated glomerular filtration rate (eGFR), alkaline phosphatase (ALP), and lactate dehydrogenase (LDH), remained stable throughout therapy.

Safety evaluations demonstrated stable clinical and biochemical profiles following [^225^Ac]Ac-PSMA-617 TAT. Figure 2 shows no Grade ≥3 hematologic or renal adverse events and no toxicity-related treatment modification, dose reduction, or discontinuation, consistent with a favorable safety profile. Hematologic and renal biomarkers also remained stable throughout therapy, with no statistically significant changes observed between baseline and follow-up values.

PSA levels before and after [^225^Ac]Ac-PSMA-617 TAT are shown descriptively in Figure 2 for responding and non-responding patients, defined by the ≥50% PSA decline criterion used to construct these groups. As the grouping and the compared variable are derived from the same metric, no inferential statistical comparison is presented for this figure to avoid circularity.

Functional status, assessed by the Barthel Index, which measures the patients’ ability to perform activities of daily living, remained preserved with a median score of 100 (100 points: the individual is completely independent in all 10 Activities of Daily Living (ADLs) and no physical assistance is required for any daily activity.) before and after TAT. This indicates that patients maintained full independence in daily living, indicating that functional independence in activities of daily living was preserved.

Regarding salivary gland uptake (SGU), PSMA-SGU on [^68^Ga]Ga-PSMA-11 PET/CT decreased by −63.7% (SUV_max_) and −45.5% (SUV_mean_) after two cycles of [^225^Ac]Ac-PSMA-617 compared with the baseline scan before the first cycle. These reductions are directionally consistent with previously reported findings, where reductions of −61% (SUV_max_) and −42.4% (SUV_mean_) were observed after one cycle [24], although the number of cycles differs (two cycles in our cohort versus one cycle). Together, these data support a reproducible reduction in salivary gland tracer uptake early during TAT. Moreover, functional swallowing/feeding difficulty was assessed by physician; no patient reported persistent difficulty, with only transient, self-resolving issues in a minority of patients. Chart review documented Grade 1 xerostomia (CTCAE) in all patients, consistent with the grade already present following prior [^177^Lu]Lu-PSMA-617 therapy, indicating no worsening attributable to [^225^Ac]Ac-PSMA-617. Changes in PSMA-PET tracer uptake reflect PSMA expression and radioligand binding in the salivary glands and are not a direct measure of salivary function. Clinical xerostomia, assessed separately by chart review (CTCAE), remained Grade 1 in all patients without worsening, and no persistent swallowing or feeding difficulty was observed. The two findings are therefore reported as separate observations, and no quantitative correlation between them was assessed.

### 2.3. PSA-Based Biochemical Response After [^225^Ac]Ac-PSMA-617 Therapy

Figure 3 presents waterfall plots showing individual patients’ changes in PSA levels after the first cycle (panel I) and after the last cycle (panel II) of [^225^Ac]Ac-PSMA-617.

After the first cycle, 9 out of 12 patients (75%) exhibited a measurable decline in PSA levels, including 6 patients (50%) with reductions of ≥30%, of whom 3 patients (25%) had reductions of ≥50%. Conversely, 3 patients (25%) showed either stable PSA levels or biochemical progression.

At the end of treatment, 8 patients (67%) maintained PSA reductions, with 5 patients (42%) showing ≥50% decreases. Notably, all 6 patients with an initial PSA decline of ≥30% maintained a decline of ≥30% at the last follow-up, and one additional patient reached a decline of ≥30% during follow-up (7/12, 58%), suggesting durability of biochemical benefit. On the contrary, 4 patients (33%) showed either stable PSA levels or biochemical progression.

The PSA kinetics offer critical insight into early therapeutic efficacy and align with molecular imaging outcomes (see Section 2.4), reinforcing the potential of TAT for disease control in patients refractory to prior [^177^Lu]Lu-PSMA therapy.

### 2.4. Molecular Imaging Response Following [^225^Ac]Ac-PSMA-617 Therapy

Molecular treatment response was evaluated using [^68^Ga]Ga-PSMA-11 PET/CT, based on percentage change from baseline in SUV_peak_ of the target lesion (the single PSMA-avid lesion with the highest SUV_peak_ at each time point. Among the cohort:

- (7/12) exhibited SUV_peak_-based partial molecular response.
- (3/12) demonstrated SUV_peak_-based stable disease.
- (2/12) showed SUV_peak_-based progressive disease.

Although 44 therapy cycles were administered, a total of 32 follow-up imaging assessments were performed.

Across the 32 [^68^Ga]Ga-PSMA-11 PET/CT follow up imaging:

- (24/32) resulted in partial molecular response,
- (4/32) in stable disease,
- (4/32) in progression.

The disease control rate (DCR) was calculated as:

- 87.5% per evaluable follow-up [^68^Ga]Ga-PSMA-11 PET/CT assessment (28/32),
- 83.3% per patient, reflecting partial response or stable disease in 10/12 patients.

Figure 4 and Figure 5 illustrate representative [^68^Ga]Ga-PSMA-11 PET/CT images, including individuals previously refractory to [^177^Lu]Lu-PSMA-617 therapy who demonstrated near-complete molecular and biochemical responses after three cycles of TAT. Others who had progressed after treatment pauses showed disease stabilization following [^225^Ac]Ac-PSMA-617 TAT. Total tumor volume (TTV) was assessed separately, as described in Section 4.3, and its relationship to PSA kinetics is presented in Figure 4 and Figure 5. Figure 4 and Figure 5 present selected illustrative cases, chosen to demonstrate representative response patterns, including cases spanning treatment pauses; they are not intended to depict the standard imaging schedule.

### 2.5. Survival Outcomes

Progression-free survival (PFS) and overall survival (OS) were analyzed using Kaplan–Meier methodology (Figure 6). During follow-up, 6 of 12 patients (50%) were alive at last contact, and 6 (50%) had died. The observed survival metrics were:

- Median PFS: 27 weeks (95% CI: 23–31 weeks)
- Median OS: 53 weeks (95% CI: 36–70 weeks)
- Probability of OS >100 weeks: 16%

Univariate analysis revealed that elevated PSA, ALP, and LDH, along with low hemoglobin, platelet count, and creatinine, were associated with poorer OS (Table 3).

These findings suggest that integrating PSA with routine blood biomarkers can offer enhanced prognostic insight and support tailored therapeutic strategies in mCRPC. Post-cycle-2 biomarker assessments were performed at approximately 12 weeks after treatment initiation (two 6-week treatment cycles). These exploratory analyses relate post-treatment (post-cycle-2) biomarker values to survival measured from the time of treatment initiation. As all patients survived to the post-cycle-2 assessment, this approach avoids survivorship bias; however, the conditional, non-baseline nature of these biomarker measurements should be taken into account when interpreting the reported associations.

Of the 12 patients, 6 deaths (events) were recorded during follow-up. PSMA PET-derived TTV was available for 9/12 patients. The remaining 3 patients were excluded from TTV-based analyses because, among their serial follow-up PET/CT scans, at least one imaging time point was performed using [^18^F]FDG PET/CT instead of [^68^Ga]Ga-PSMA-11 PET/CT, due to a deviation from the standard imaging protocol. As TTV quantification relies on consistent PSMA-avid volumetric segmentation across sequential time points, the presence of a non-PSMA (FDG) scan within the imaging series precluded reliable longitudinal TTV assessment for these patients. In contrast, per-patient molecular response categorization (PR/SD/PD; Section 2.4) was based on the available [^68^Ga]Ga-PSMA-11 PET/CT scans at each patient’s respective follow-up time points and was therefore not affected by this issue. Table 4 summarizes the Firth-penalized hazard ratio (per 1-SD increase) and bootstrapped 95% CI for each biomarker.

Two biomarkers, PSA and ALP, showed bootstrapped 95% CIs that excluded the null value of 1, indicating a statistically detectable association with OS in this cohort. Both findings were concordant with the primary Kaplan–Meier/log-rank analysis (Table 3), in which PSA (*p* = 0.002) and ALP (*p* = 0.012) were also significant, showing concordant results across two statistical approaches applied to the same cohort, which increases confidence that these associations are not an artifact of a single analytical choice, though this concordance does not constitute independent validation given the shared dataset.

Four biomarkers, hemoglobin, platelets, creatinine, and LDH, were significant in the median-dichotomized log-rank analysis (*p* < 0.001, *p* < 0.001, *p* = 0.003, and *p* = 0.014, respectively) but did not reach significance in the Cox model (bootstrapped 95% CI 0.11–1.04, 0.48–6.49, 0.10–1.63, and 0.78–12.23, respectively, all crossing 1). No other biomarker (eGFR, TTV) reached statistical significance in either analysis.

### 2.6. Pooled Quantitative Analysis: Contextualizing [^225^Ac]Ac-PSMA-617 Efficacy and Safety Across Studies

To place our retrospective findings within a broader clinical framework, our literature search (original and updated, Supplementary Figure S2) identified 11 studies eligible for the descriptive safety/toxicity synthesis. Of these, one study did not report a discrete PSA-decline outcome and was therefore not eligible for the pooled quantitative analysis [11]; this study reported a composite outcome (objective radiologic response or PSA decline in 9/11 evaluable patients) rather than a discrete PSA ≥50% metric (see Table 6 footnote). For all other studies, a PSA decline reported as ≥50% or as more than 50% was treated as PSA50 response [2,12]. This harmonization rule was applied uniformly to all studies; the distinction between “>50%” and “≥50%” would likely have a negligible numerical impact for most individual patients, and its influence on the pooled estimate was tested in a sensitivity analysis (see below). One study [11] is reported descriptively in Table 6 alongside the other 10 eligible studies, but was not included in the discrete pooled quantitative analysis, which encompassed the remaining 10 studies and 745 patients [2,7,10,12,13,14,15,16,17,25]. This primary analysis yielded a pooled effect size of 0.60 (95% CI: 0.51–0.68; I^2^ = 65.32%). Given that one study [14] contributed disproportionately to between-study heterogeneity, we additionally performed a sensitivity analysis excluding this study (9 studies, 726 patients), which yielded a pooled effect size of 0.62 (95% CI: 0.55–0.70; I^2^ = 55.7%). The primary and sensitivity estimates (0.60 vs. 0.62) were similar, with largely overlapping confidence intervals, indicating that the pooled estimate is not materially influenced by the inclusion or exclusion of this study. The between-study heterogeneity, however, decreased from I^2^ = 65.3% to 55.7%, and remained substantial. We also performed a sensitivity analysis excluding the two studies that reported PSA response as a decline of more than 50% ([12] and [2]; 8 studies, 679 patients). The pooled effect size was 0.62 (95% CI: 0.52–0.71; I^2^ = 67.6%), indicating that the harmonization rule does not materially influence the pooled estimate. Excluding the study [17] (the only study using [^225^Ac]Ac-PSMA-I&T rather than [^225^Ac]Ac-PSMA-617) gave 0.60 (95% CI: 0.52–0.69; 9 studies, 731 patients), and in a leave-one-out analysis, the pooled estimate ranged from 0.57 to 0.62 across all single-study exclusions. Risk-of-bias assessment (Supplementary Table S2) indicated a low risk of bias across all 10 included studies (6/6 in eight studies, 5/6 in two studies), supporting the reliability of the pooled effect estimate. A pooled quantitative analysis of median PFS and OS was not performed because hazard ratios (and their associated variances) were not available in the pooled data. Figure 7 shows a comparison of the results from our retrospective cohort study (n = 12) with the estimates from the pooled quantitative analysis. We present our cohort’s outcome and the pooled quantitative estimate from the literature descriptively, side-by-side in a forest plot, without statistically combining them into a new pooled estimate.

To visualize the individual contribution of each study to the primary pooled estimate, Figure 8 presents the corresponding forest plot for the ten included studies, together with a funnel plot assessing potential publication bias.

#### 2.6.1. Pooled Efficacy Outcome

These results revealed a pooled PSA response rate of 0.60 (≥50% decline). The result supports the clinical relevance of [^225^Ac]Ac-PSMA-617, particularly in pretreated mCRPC patients, including those refractory to [^177^Lu]Lu-PSMA-617 therapy. The pooled PSA response rate aligns with our findings, though our cohort’s lower response may reflect more advanced disease burden prior [^177^Lu]Lu-PSMA RLT.

#### 2.6.2. Study-Level Insights

Table 5 presents current strategies for the prevention of TAT-induced xerostomia, while Table 6 summarizes the key outcome measures in selected clinical studies (n = 11).

## 3. Discussion

Metastatic castration-resistant prostate cancer represents a major cause of cancer-related mortality among men and remains a significant therapeutic challenge despite advances in systemic treatments. Radioligand therapy with [^177^Lu]Lu-PSMA-617 has demonstrated significant clinical efficacy in patients with mCRPC following prior lines of systemic treatment including hormone therapy, and chemotherapy; however, a subset of patients unfortunately exhibits limited or no response in PSA reduction [11,13,17]. In our department, [^225^Ac]Ac-PSMA-617 TAT has been administrated to a selectively chosen patient cohort following multidisciplinary tumor board evaluation. This approach used to improve therapeutic efficacy. Within the framework of our retrospective analysis, we assessed targeted alpha therapy and directly compared its therapeutic outcomes with those of [^177^Lu]Lu-PSMA-617 using hematological and renal function biomarkers. While PSA remains a standard biomarker for assessing therapeutic response in mCRPC, its limitations are increasingly evident, particularly in patients receiving beta or alpha therapies where discordance between PSA kinetics and clinical or imaging response is not uncommon [27]. Recent studies have emphasized incorporating additional biomarkers to achieve a more comprehensive assessment of treatment efficacy. Biochemical markers such as alkaline phosphatase (ALP) and gamma-glutamyl transferase (GGT) have demonstrated correlations with treatment outcomes, particularly in patients treated with [^177^Lu]Lu-PSMA-617 [28]. They mentioned that lower GGT and ALP at treatment start were significantly associated with a lower risk of progression during [^177^Lu]Lu-PSMA-617 therapy and GGT > 31 U/L at treatment start remained a statistically significant predictor, associated with 1.5 times higher risk of progression. A multicenter trial including 61 patients pretreated with abiraterone/enzalutamide (75.4%) and docetaxel/cabazitaxel (68.9%) who received three cycles of PSMA-RLT depicted that the levels of basal Hb were able to predict survival of patients. Similarly, normal pre-treatment Hb levels were predictive for a ≥ 50% PSA decline during therapy, while lower pre-treatment Hb levels were associated with a lack of PSA reduction [29]. However, another study did not confirm this finding, possibly caused by the relatively low patient number included in this trial [30]. Moreover, the presence of high LDH levels was associated with poor OS in several trials assessing its predictive impact during RLT [31,32,33]. Interestingly, LDH kinetics within two to three months during therapy have been reported as a predictive biomarker in a retrospective trial involving 137 patients [31]; however, another trial did not confirm this finding [30]. Integrating these biochemical biomarkers with PSA kinetics could offer a more comprehensive framework for evaluating therapeutic efficacy and optimizing treatment selection in mCRPC. Accordingly, PSMA-PET TTV and SUV metrics, PSA kinetics, and routine blood biomarkers were considered primary translational endpoints in this analysis, providing complementary insight into early biological response patterns under [^225^Ac]Ac-PSMA-617 therapy. Based on our pooled quantitative analysis, median PFS and OS could not be reliably estimated due to the unavailability of hazard ratios and their associated variances. Nevertheless, [^225^Ac]Ac-PSMA-targeted alpha therapy (TAT) demonstrated substantial therapeutic benefit in pretreated mCRPC patients, with reported median OS ranging from 7.7 to 15.5 months, median PFS from 3.1 to 15.2 months, and PSA response rates between 26% and 88% (Table 6). However, notable safety concerns were observed, particularly xerostomia (77%) grade I-II and hematologic toxicities (4–37%), whereas renal toxicity remained comparatively low (up to 7%). Collectively, these findings highlight a favorable efficacy profile but emphasize the importance of carefully weighing safety considerations in clinical decision-making. We evaluated the efficacy and outcomes of [^225^Ac]Ac-PSMA-617 in 12 mCRPC patients using biochemical and molecular tumor responses, DCR, and survival analysis. A PSA decline was observed in 75% of patients after the first cycle, with a PSA decline of ≥50% in 25%. At the last follow-up, 67% of patients had a PSA decline, with 42% showing a ≥50% reduction, while 25% had progressive disease (Figure 3). The median PFS was approximately 27 weeks (6.3 months), and the median OS was around 53 weeks (12.4 months) (Figure 6). No Grade ≥3 hematologic or renal adverse events were documented and no toxicity-related treatment modifications occurred, consistent with minimal hematologic and renal toxicity; this clinical observation was further supported by the absence of statistically significant changes in ALP, creatinine, eGFR, hemoglobin, LDH, platelet, or WBC levels between pre- and post-therapy (Figure 2).

The observed biomarker changes after TAT can be contextualized within established alpha-particle radiobiology and PSMA-targeting physiology. Alpha particles deliver high-LET radiation that induces densely clustered DNA double-strand breaks, leading to rapid apoptotic and necrotic tumor cell death [34]. This mechanism aligns with the early declines in PSA, LDH, and ALP observed in our cohort, as these biomarkers reflect tumor metabolic activity, cellular turnover, and osteoblastic metastatic burden.

Variability in biomarker response may be influenced by the tumor sink effect, whereby patients with extensive metastatic disease sequester a larger fraction of PSMA-targeted radioligand, reducing off-target uptake in normal tissues [35]. This phenomenon has been described for both β- and α-emitting PSMA ligands and may explain the heterogeneity in hematologic and renal changes in our cohort. Patients with lower tumor burden may receive proportionally higher non-target uptake to kidneys, contributing to eGFR fluctuations.

Salivary-gland toxicity is mechanistically linked to physiological PSMA expression in acinar cells, resulting in unintended radiopharmaceutical accumulation [36]. Alpha particles may cause irreversible DNA damage in salivary tissue, consistent with the xerostomia.

Integrating these established mechanistic insights provides a biologically coherent interpretation of the PSA, LDH, ALP, hematologic, and renal biomarker changes observed in this cohort.

When comparing our findings with the pooled quantitative analysis, both observed favorable PSA responses (Figure 7 and Figure 8). However, our study shows a lower PSA response rate (42% with ≥50% decline) compared to the pooled quantitative analysis (60%). This discrepancy may be attributed to differences in patient populations, as our cohort included patients refractory to previous [^177^Lu]Lu-PSMA-617 therapy, which may have contributed to the lower biochemical response observed. Given the small number of studies included in the pooled quantitative analysis (n = 10), the assessment of publication bias using Begg’s and Egger’s tests should be interpreted with caution, as these tests have limited statistical power when the number of included studies is small.

Regarding safety, the pooled quantitative analysis highlighted xerostomia as a significant concern (Table 6). In our cohort, however, xerostomia remained Grade 1 (CTCAE) without worsening, despite the reduction in salivary gland PSMA uptake on PET after two cycles compared with baseline. Tracer uptake and clinical xerostomia are distinct endpoints, and the former cannot be used to infer the latter. In addition, renal toxicity remained minimal, with no Grade ≥3 renal adverse events or renal-related treatment modifications documented, consistent with the observed stability of eGFR and creatinine levels.

Our results align closely with previously reported findings [7], where a significant proportion of patients experienced PSA declines exceeding 50%. Our findings are comparable to the 39% reported in a cohort [2] that likewise consisted largely of heavily pretreated patients, more than half of whom were refractory to prior [^177^Lu]Lu-PSMA-617. Additionally, our cohort had previously received [^177^Lu]Lu-PSMA-617 therapy, whereas only two patients in a comparable study [7] had prior radioligand therapy before TAT, making direct comparisons of treatment resistance challenging.

Similarly, a previous study [17] reported that 50% of patients had PSA declines following a single cycle of [^225^Ac]Ac-PSMA-I&T. [^225^Ac]Ac-PSMA-617 and [^225^Ac]Ac-PSMA-I&T share the same molecular target and radionuclide, differing only in chelator/linker chemistry; both were therefore considered within the predefined scope of [^225^Ac]Ac-PSMA TAT for this analysis. A sensitivity analysis excluding this study changed the pooled PSA-response effect size from 0.60 (95% CI: 0.51–0.68; ten studies) to 0.60 (95% CI: 0.52–0.69; nine studies), confirming that the pooled estimate is not driven by inclusion of his ligand-heterogeneous study [17]. Higher PSA response rates (65% with ≥50% reduction) have also been reported, possibly due to differences in disease stage, metastatic sites, tumor burden, and biodistribution [13].

PET/CT imaging using PSMA peptide remains the preferred modality for evaluating treatment efficacy in RLT and TAT. Our study incorporated TTV as a complementary, continuous quantitative imaging biomarker alongside SUV_peak_-based molecular response classification. Post-[^225^Ac]Ac-PSMA-617 therapy assessment using [^68^Ga]Ga-PSMA-11 PET/CT demonstrated a SUV_peak_-based partial response in 58% of patients, with 42% achieving ≥ 50% PSA declines. The concordance between early reductions in TTV and PSA levels suggests that PSMA PET-derived volumetric parameters reflect biologically meaningful tumor response to [^225^Ac]Ac-PSMA-617 TAT. Although our study was not statistically powered to formally test the association between TTV, PSA, and OS in combination, a qualitative concordance between TTV and PSA response was observed in a subset of patients (n = 4, Figure 4 and Figure 5), in whom both parameters declined or increased in parallel over the course of therapy. Given that PSA response was significantly associated with OS in our cohort, this concordant behavior raises the hypothesis that TTV dynamics may likewise relate to survival outcomes, although this could not be formally tested given the limited sample size of our pilot cohort. This observation is in line with a previous report [37], in which TTV was similarly described as an imaging biomarker independent of PSA in patients undergoing [^225^Ac]Ac-PSMA-I&T; however, a significant prognostic value of TTV changes could likewise not be established in that small cohort (n = 13), and the authors explicitly noted that this issue would need to be addressed by larger, prospective study designs. In contrast, larger cohorts of patients treated with [^177^Lu]Lu-PSMA-617 have shown that PSMA PET-derived TTV can independently predict outcome [38], supporting the biological plausibility of TTV as a prognostic imaging biomarker once adequately powered data become available. Taken together, these findings suggest that the combined evaluation of imaging-derived tumor burden and biochemical response may hold prognostic value beyond what either parameter can demonstrate in isolation in small retrospective cohorts, a hypothesis that warrants confirmation in larger, prospective studies specifically designed to test this association.

A recent large-scale study involving 488 patients reported a median OS of 15.5 months and PFS of 7.9 months [15], while our study found an OS of 12.4 months and a PFS of 6.3 months. Variations in OS may stem from differences in patient characteristics and prior treatments. Unlike the previous multicenter study [15], we incorporated blood biomarker analysis (Table 2), revealing significant correlations between treatment outcomes and specific biomarkers.

Our study represents a potential first step in evaluating the role of blood biomarker results for TAT efficacy assessment, warranting further investigation. Additionally, the qualitative concordance observed between TTV and PSA changes in individual patients (Figure 4 and Figure 5) points to a promising avenue for future research: TTV derived from [^68^Ga]Ga-PSMA PET/CT could serve as a non-invasive surrogate for absorbed-dose estimation, offering a practical alternative to the technically challenging post-treatment Single-Photon Emission Computed Tomography (SPECT) based dosimetry currently limited by suboptimal image quality. Formally testing this hypothesis in a dedicated dosimetry cohort represents a natural and valuable next step.

Our study indicated that the univariate blood biomarker results (Table 3) may contribute to treatment efficacy stratification. Higher PSA, ALP, and LDH levels after two cycles of [^225^Ac]Ac-PSMA-617 were associated with shorter OS. Elevated LDH and ALP after two cycles of therapy likely reflect aggressive, metabolically active disease and bone metastasis burden, respectively, which may reduce effective tumor control by TAT and explain shorter OS in these patients. Furthermore, lower hemoglobin, platelet, and creatinine levels were associated with poorer survival outcomes. Although eGFR showed a trend toward an association with OS, this finding did not reach statistical significance. The associations observed between these biomarkers and OS suggest that future studies could benefit from applying multivariate survival models, similar to those previously used for prior treatment variables [15]. Such approaches may further refine prognostic assessment and support more personalized management of patients undergoing [^225^Ac]Ac-PSMA-617 therapy.

These clinical outcomes are consistent with the univariate biomarker associations described above. Formal testing of whether the combined biomarker framework offers incremental prognostic value beyond PSA alone was beyond the scope of this exploratory, hypothesis-generating analysis. Patients with lower post-cycle-2 LDH, ALP, and PSA, biomarkers typically associated with less aggressive disease biology, were more likely to achieve meaningful PSA declines, TTV reductions, and longer OS/PFS. Conversely, patients with elevated LDH or ALP, or reduced hematologic reserve, demonstrated poorer survival outcomes, aligning with the prognostic implications of these biomarkers. The concordance between biochemical markers and clinical endpoints supports the utility of integrating PSA, LDH, ALP, hemoglobin, creatinine, and eGFR into early response stratification frameworks for [^225^Ac]Ac-PSMA-617 therapy.

This exploratory analysis (Table 4) identified PSA and ALP as biomarkers showing a statistically detectable association with OS following [^225^Ac]Ac-PSMA-617 TAT in this heavily pretreated, [^177^Lu]Lu-PSMA non-responsive cohort. These two associations were consistent between the median-dichotomized Kaplan–Meier/log-rank analysis and the Firth-penalized, bootstrapped Cox regression; as both analyses were performed on the same 12 patients (6 deaths), this concordance reflects internal consistency across modeling approaches rather than independent validation, though it reduces the likelihood that either finding is an artifact of a single analytical choice. Given the small sample size, these survival associations should be regarded as exploratory and hypothesis-generating rather than confirmatory. This concordance is also biologically plausible: elevated ALP and PSA are established markers of bone/hepatic metastatic burden and overall disease aggressiveness in mCRPC, and have been repeatedly linked to inferior survival across chemotherapy and radioligand therapy settings [39,40].

In contrast, hemoglobin, platelets, and creatinine were significant in the dichotomized log-rank analysis but not in the Cox model. This discrepancy is informative rather than contradictory, and likely reflects the sensitivity of small-sample, median-split analyses to the specific threshold chosen: with only 12 patients, a median split creates two strata of just 6 patients each, and the log-rank result can be strongly influenced by 1 or 2 patients near the cut-point. The Cox model, which uses the full distribution of values rather than a single dichotomization, did not detect the same magnitude of association for these three markers, suggesting their apparent prognostic value in Table 3 may be, at least in part, threshold-dependent rather than reflecting a robust dose–response relationship across the full biomarker range. Notably, hemoglobin’s bootstrapped interval (0.11–1.04) approached but did not cross the threshold for significance, indicating a borderline signal that may still warrant attention in a larger cohort. We report all results transparently rather than selecting the more favorable ones, and recommend that hemoglobin, platelet, and creatinine associations be interpreted with additional caution relative to PSA and ALP until validated in a larger cohort.

Given the retrospective design and limited cohort size (n = 12, 6 events), a multivariable model combining biomarkers was not statistically supportable and was intentionally not performed, in line with recognized statistical guidance for survival analysis in small samples [41]. Firth-penalized Cox regression was adopted a priori as the primary analytical approach, and bootstrapped confidence intervals were used throughout to provide a more conservative and transparent estimate of uncertainty than single point estimates alone. This approach explicitly surfaced a known small-sample artifact, quasi-complete separation during resampling, which was systematically addressed through penalized estimation rather than omitted from reporting [42].

These results should be interpreted as hypothesis-generating rather than confirmatory. The wide confidence intervals, particularly for TTV, where imaging data were unavailable in 3/12 patients, further illustrate the inherent limitation of drawing firm prognostic conclusions from a cohort of this size. Validation of PSA and ALP as prognostic markers, and clarification of the hemoglobin, platelet, and creatinine discrepancies, in a larger prospectively collected cohort is warranted before these markers could be proposed as clinical decision-support tools in this treatment setting.

Notably, the Barthel Index remained unchanged following both [^177^Lu]Lu-PSMA RLT and [^225^Ac]Ac-PSMA TAT, suggesting that functional independence was preserved under both therapies (Table 1). This reinforces the potential of sequential TAT to maintain quality of life while providing therapeutic benefits.

In the illustrative patients shown in Figure 4 and Figure 5, salivary gland uptake appeared to vary between successive therapy cycles, with a decrease on the follow-up PET/CT. This qualitative pattern, which was not assessed at the cohort level, is compatible with a tumor sink effect, in which changes in tumor burden redistribute the tracer to normal organs, and may serve as an indirect marker of treatment response. Whether such changes in uptake translate into clinical xerostomia was not assessed and warrants careful monitoring.

This study has some limitations inherent to its retrospective, single-center design and small sample size. The requirement for complete follow-up PET/CT imaging after every two treatment cycles resulted in the exclusion of four patients from the molecular imaging response analysis, as these patients transferred their post-treatment imaging follow-up to other institutions and were therefore lost to imaging follow-up at our center. This exclusion was administrative in nature, related to the single-center design of the study, rather than driven by clinical deterioration or disease progression, and is therefore unlikely to have systematically biased the observed response rates toward a more favorable outcome. Nonetheless, missing data of any kind can reduce statistical power and limit the generalizability of the findings, underscoring the value of multi-center prospective designs with centralized imaging follow-up in future studies.

The study is framed as a multimodal biomarker-focused exploratory analysis integrating quantitative PSMA-PET parameters with blood biomarkers to support early response interpretation and hypothesis generation. Future multi-center studies with larger cohorts and prospective designs will be essential to further validate these findings and enhance their applicability to broader patient populations.

It’s important to emphasize that our cohort represented a particularly challenging clinical context. All patients had previously undergone multiple cycles of [^177^Lu]Lu-PSMA-617 (Figure 1), finally resulting in biochemical non-responsiveness and signs of progressive disease. At the time of transition to [^225^Ac]Ac-PSMA-617 TAT, several patients already exhibited early symptoms of xerostomia highlighting not only the cumulative treatment burden but also the clinical complexity of decision-making in this setting. The initiation of TAT in these patients was far from straightforward; it involved balancing the potential therapeutic benefit of high-LET alpha particle therapy against the known and anticipated risks of dose-limiting toxicity. This underscores the need for tailored interventions, informed consent, and quality-of-life–focused treatment planning, especially when patients are navigating refractory disease states. It is important to note that severity grading is inconsistently reported across pooled studies often limited to Common Terminology Criteria for Adverse Events (CTCAE) Grade 1–2 without longitudinal follow-up. In our cohort, all affected patients remained free of high-grade (CTCAE Grade ≥3) xerostomia and reported no persistent swallowing or feeding difficulty, suggesting a predominance of mild-to-moderate (Grade I) toxicity. Thus, this comparison likely reflects a worst-case prevalence scenario, rather than a direct severity match. The elevated xerostomia rate in our cohort likely stems from cumulative radioligand exposure, low tumor burden (tumor sink effect), and prior [^177^Lu]Lu-PSMA exposure. These observations underscore the importance of toxicity-aware dosing and personalized mitigation approaches. In response to this challenge, we implemented external cooling (ice packs) during radioligand infusion. Despite its practicality and favorable tolerability, recent evidence [26] demonstrated limited efficacy in reducing salivary uptake. To enhance protective efforts, we now plan to introduce prophylactic oral vitamin C, which has demonstrated promising SUV_mean_ reductions ranging from 26–52% in prospective PSMA-PET/CT studies. These efforts reflect an evolving, patient-centered strategy to reduce glandular toxicity, preserve quality of life, and refine supportive care protocols. Looking ahead, future therapeutic planning in mCRPC should prioritize clinical frameworks that better navigate the trade-offs between efficacy and tolerability especially for patients with prior radioligand exposure and symptomatic toxicity. Our findings highlight the need for tools that integrate disease burden, prior [^177^Lu]Lu-PSMA exposure, and functional salivary gland status into predictive algorithms or TAT suitability scores. Personalized models could enable risk stratification for severe xerostomia and help optimize dosing schedules accordingly. Moreover, integration of validated patient-reported outcome instruments would allow longitudinal tracking of xerostomia severity and reversibility, providing a foundation for survivorship planning and therapeutic adaptation.

## 4. Materials and Methods

### 4.1. Patients and Ethics

This retrospective study included consecutive patients with mCRPC who received [^225^Ac]Ac-PSMA-617 therapy after demonstrating resistance to prior treatments. Patients were considered treatment refractory if they no longer responded to androgen deprivation therapy (ADT), chemotherapy, radiotherapy, and multiple cycles of [^177^Lu]Lu-PSMA-617 RLT (median (min–max): 4 (2–8) cycles). Other resistance was determined by a sustained increase in serum PSA levels, progression of existing disease, or the appearance of new metastases despite castrate testosterone levels. To be included in the analysis, patients had to have complete follow-up data available at least 6 weeks after each cycle. Eligibility for [^225^Ac]Ac-PSMA-617 TAT required progression on or resistance to [^177^Lu]Lu-PSMA-617 RLT, or the presence of extensive metastasis despite prior treatments.

Supplementary Figure S1 summarizes the patient flow for this analysis. Of 16 patients with mCRPC treated with [^225^Ac]Ac-PSMA-617 after inadequate response to [^177^Lu]Lu-PSMA-617 RLT, 4 were excluded from the analysis because they were missing the PET imaging that was scheduled to be performed after every two treatment cycles per protocol. Therefore, we evaluated the data of 12 patients, who together received a total of 2–6 treatment cycles.

Treatment decisions were approved by a tumor board after a comprehensive review of each patient’s medical history and current status. The study was conducted at the Department of Nuclear Medicine, Philipps University Hospital, Marburg, Germany, between July 2021 and January 2024.

Treatment was administered on a compassionate use basis in accordance with the German Medical Products Act (Arzneimittelgesetz—AMG §13 (2b)). To be eligible, patients must have had disease progression despite other treatments such as chemotherapy, radiotherapy, or second-generation anti-androgen therapy, or the tumor board determined that these interventions were contraindicated. All patients were informed of the experimental nature of [^225^Ac]Ac-PSMA-617 therapy and signed consent forms.

This retrospective study was approved by the Ethics Committee of the School of Medicine at Philipps University of Marburg (Ethics vote reference number: 24-109 RS, approval date: 25 April 2024).

### 4.2. [^225^Ac]Ac-PSMA-617 Administration

The PSMA-617 peptide was labeled with [^225^Ac]AcCl_3_ (ITM Isotope Technologies, Garching, Germany), and after radiosynthesis and quality control, it was administered intravenously. The administered activity was 100 kBq/kg of [^225^Ac]Ac-PSMA-617, an activity previously proposed [11] as a balanced amount to enhance therapeutic efficacy while minimizing adverse effects. Details of the labeling process are described in Supplementary Section S1.

To potentially reduce salivary gland perfusion, radiopharmaceutical uptake, and toxicity, patients’ salivary glands were covered with cool packs 30 min before and up to 4 h after radiopharmaceutical administration. In the event of post-injection nausea, patients were treated with Granisetron.

In compliance with the German Radiation Protection Act, [^225^Ac]Ac-PSMA-617 therapy was administered on an inpatient basis, with patients remaining in the nuclear medicine ward for at least 48 h post-administration before being discharged when deemed medically appropriate. A minimum of two cycles of therapy were scheduled, administered at 6-week intervals. Subsequent dosage, cycle number, and interval were adjusted individually based on the disease burden, patient condition, and logistical factors.

### 4.3. Safety, Efficacy, and Treatment Outcome Evaluation

#### 4.3.1. Safety Monitoring

Safety was evaluated based on laboratory data, including blood biomarkers (hemoglobin, white blood cell count, platelet count, creatinine level, estimated glomerular filtration rate (eGFR), alkaline phosphatase (ALP), lactate dehydrogenase (LDH), and renal function tests). These were assessed at baseline, after each subsequent cycle, and during follow-up. Bone marrow, and renal toxicities—common side effects of this treatment modality—were graded using laboratory data. Toxicity was graded retrospectively according to CTCAE v5.0 criteria based on chart review. A minority of patients had Grades 1–2 hematologic laboratory abnormalities; no Grade ≥3 hematologic or renal adverse event was documented, and no treatment modification, dose reduction, or discontinuation due to toxicity occurred in this cohort. As this was a retrospective chart-based assessment rather than a prospective, protocol-driven toxicity evaluation with scheduled CTCAE grading at each visit, mild or asymptomatic laboratory abnormalities not explicitly documented by the treating physician may have been underreported; the absence of higher-grade toxicity should therefore be interpreted with this limitation in mind.

#### 4.3.2. Salivary Gland Uptake

Quantitative parameters, including SUV_max_, and SUV_mean_, were extracted for the salivary glands as organs at risk (OAR) through segmentation of [^68^Ga]Ga-PSMA-11 PET/CT images. Segmentation was performed using HERMES software (HERMES Medical Solutions, Sweden), which enabled precise delineation of salivary glands for accurate measurement and analysis.

#### 4.3.3. Renal Function

Each patient underwent dynamic renal scintigraphy using [^99m^Tc]Tc-MAG3 prior to each cycle to detect any potential urinary tract obstruction [43].

#### 4.3.4. Functional Status

The Barthel Index was calculated to evaluate the patient’s functional status at baseline and after treatment. This index assesses a patient’s ability to perform daily activities, with scores ranging from 0 (complete dependence) to 100 (complete independence) [44]. The index was administered by trained clinicians at our institution.

#### 4.3.5. Efficacy and Treatment Outcome Evaluation

It was characterized using five endpoints:

1. Biochemical response and progression were assessed according to the Prostate Cancer Working Group 3 (PCWG3) criteria [45]. A PSA decline of ≥30% from baseline was considered a positive sign of therapeutic efficacy, and a decline of ≥50% was defined as a biochemical partial response. Biochemical disease progression was defined, per PCWG3, as an increase in PSA of ≥25% and an absolute increase of ≥2 ng/mL above the nadir value (or above baseline, if no decline had occurred), confirmed by a second measurement obtained ≥3 weeks later. Serial PSA measurements were obtained before every treatment cycle (median: 6 (3–9 weeks) every 6 weeks) and concurrently with each [^68^Ga]Ga-PSMA PET/CT assessment, performed after every two treatment cycles. To minimize the risk of misclassifying early, non-specific PSA fluctuations as true disease progression in this heavily pretreated, [^177^Lu]Lu-PSMA-617-refractory population, disease progression was defined using a dual, concordant criterion, assessed at these joint PSA/PET time points: a PSA rise, confirmed by a second measurement obtained ≥3 weeks apart from the serial pre-cycle measurements, together with concurrent molecular progression on [^68^Ga]Ga-PSMA PET/CT obtained at the same visit (per item 2). An isolated PSA rise without concordant PET evidence at the corresponding assessment was never used alone to assign disease progression.
2. Molecular tumor response after every two cycles of TAT was categorized based on percentage change from baseline in SUV_peak_ of PSMA-avid target lesions. SUV_peak_, rather than the lean-body-mass-corrected SUL_peak_ used a previous study [2], was used because lean-body-mass correction was not part of the segmentation workflow applied in this study; this response framework therefore represents a study-specific, exploratory adaptation of that approach [2], rather than a direct replication of their criteria. Molecular partial response was defined as a >30% decrease in SUV_peak_ and molecular progressive disease as a >30% increase in SUV_peak_, matching the previously used threshold values [2]; molecular stable disease was defined as a change within these thresholds. The target lesion was defined, following the previously adopted PERCIST 1 approach [2], as the single PSMA-avid lesion with the highest SUV_peak_ (rather than the lean-body-mass-corrected SUL_peak_ used in that study [2]) at each imaging time point, identified using a 1-cm^3^ spherical volume of interest; the target lesion was not required to be the same anatomical lesion at baseline and follow-up. Lesions with uptake attributable to physiological background were excluded.
3. In addition to lesion-level SUV_peak_-based response categorization, PSMA PET-derived total tumor volume (TTV) was quantified as a separate, continuous quantitative imaging biomarker using semi-automated threshold-based (SUV ≥ 4.0) segmentation, and was evaluated independently for its association with PSA kinetics (Figure 4 and Figure 5) and overall survival (Table 3 and Table 4).
  PET/CT images were acquired using an injected activity of 2–2.5 MBq/Kg (approximately 150–200 MBq) [^68^Ga]Ga-PSMA-11 with an uptake time of 60 min and manual VOI corrections were applied when automated segmentation required adjustment.
  TTV was quantified using a semi-automated, threshold-based (SUV ≥ 4.0) segmentation method. SUV_max_ and SUV_mean_ were determined for tumor lesions and salivary glands using defined volume of interests. All segmentation and quantitative image analyses were performed by HERMES software V1.1.0 (Hermes Medical Solutions, Stockholm, Sweden).
4. Disease control rate (DCR) was calculated at two levels: at the patient level, by dividing the number of patients exhibiting a partial response or stable disease as their best molecular imaging response by the total number of patients; and at the assessment level, by dividing the number of evaluable follow-up [^68^Ga]Ga-PSMA-11 PET/CT assessments demonstrating partial response or stable disease by the total number of evaluable follow-up assessments performed across cycles and patients. [^68^Ga]Ga-PSMA-11 PET/CT was scheduled after every two treatment cycles; additional assessments were performed at the treating physician’s discretion, including around treatment pauses, yielding 32 evaluable follow-up PET/CT assessments across 44 administered cycles.
5. PFS was defined as the time from the first TAT administration to documented disease progression or death from any cause, whichever occurred first. Disease progression was determined using the study-specific concordant biochemical and molecular criteria described in item 1 (a confirmed PSA rise together with concurrent molecular progression on [^68^Ga]Ga-PSMA-11 PET/CT). Patients without an event were censored at the date of their last follow-up. OS was defined as the interval between the first TAT administration and the patient’s death or the date of last clinical visit. In all patients who died during follow-up (6/12), death occurred after documented disease progression, meaning the PFS and progression-only endpoints coincide numerically in this cohort.

### 4.4. Pooled Quantitative Analysis

To contextualize our findings within previously published data, we conducted a pooled quantitative analysis of PSA response rates (PSA decline ≥50%) in patients with mCRPC treated with [^225^Ac]Ac-PSMA TAT, applying statistical methods similar to those used in a meta-analysis, rather than a formal meta-analysis. A comprehensive clinical literature search was performed in PubMed, Scopus, and Google Scholar to identify relevant studies published up to 15 July 2026. The following search strategy, adapted to the syntax of each database, was applied:

PubMed: (“Actinium-225”[tiab] OR “225Ac”[tiab] OR “Ac-225”[tiab] OR “Actinium 225”[tiab]) AND (“PSMA-617”[tiab] OR “PSMA I&T”[tiab] OR “PSMA-I&T”[tiab] OR “prostate-specific membrane antigen”[tiab] OR “PSMA”[tiab]) AND (“targeted alpha therapy”[tiab] OR “targeted alpha-therapy”[tiab] OR “alpha therapy”[tiab] OR “alpha-particle therapy”[tiab] OR “alpha emitter”[tiab] OR “radioligand therapy”[tiab] OR “radionuclide therapy”[tiab] OR “TAT”[tiab]) AND (“prostate cancer”[tiab] OR “Prostatic Neoplasms”[mesh] OR “castration-resistant”[tiab] OR “mCRPC”[tiab] OR “CRPC”[tiab]).

Scopus: TITLE-ABS-KEY((“Actinium-225” OR “225Ac” OR “Ac-225”) AND (“PSMA-617” OR “PSMA I&T” OR “PSMA-I&T” OR “prostate-specific membrane antigen” OR “PSMA”) AND (“targeted alpha therapy” OR “alpha therapy” OR “alpha-particle therapy” OR “alpha emitter” OR “radioligand therapy” OR “radionuclide therapy” OR “TAT”) AND (“prostate cancer” OR “castration-resistant” OR “mCRPC” OR “CRPC”)).

Google Scholar: (“225Ac-PSMA-617” OR “Actinium-225 PSMA” OR “225Ac-PSMA”) targeted alpha therapy prostate cancer; a complementary query, (“225Ac-PSMA-I&T” OR “Actinium-225 PSMA-I&T”) alpha therapy prostate cancer, was used to additionally capture PSMA-I&T-based studies, given the more limited Boolean syntax supported by this database.

No language restriction was applied. An updated search using the identical strategy across all three databases was performed on 15 September 2026 to capture records published since the original search. The literature search was performed independently by two reviewers; study selection and data extraction were performed independently by both reviewers, with disagreements resolved by consensus. Inclusion criteria encompassed prospective and retrospective studies reporting at least one clinical outcome, including OS, PFS, PSA response, and treatment-related toxicities such as xerostomia, hematologic toxicity, and renal toxicity. The complete literature search and study-selection process is summarized as a PRISMA flow diagram in Supplementary Figure S2 (Section S4). Study-level risk of bias was assessed independently for each included study using a modified Newcastle–Ottawa Scale adapted for single-arm cohort studies (Selection and Outcome domains only, maximum 6 stars), reported in Supplementary Table S2 (Section S5).

Relevant data were extracted from each study. For each study, the numerator was the number of patients with a best PSA decline of ≥50%, and the denominator was the number of patients evaluable for PSA response. For the pooled analysis, only PSA-based outcomes were extracted; imaging-based responses were not used. Where a study did not report this count directly [16], it was derived from the PSA data reported in the publication. Response thresholds and assessment time points were extracted as reported by each study and verified against the original publications (see Table 6 footnote). A PSA decline reported as ≥50% or as more than 50% was treated as PSA50 response; this harmonization rule was applied uniformly to all studies. A random-effects model using the Restricted Maximum Likelihood (REML) estimation method was applied to calculate the pooled effect size (proportion of events, prop) with standard errors (se), accounting for potential heterogeneity among studies. Heterogeneity was assessed using I^2^, and the statistical significance of the pooled effect was evaluated using the Z-test. Publication bias was assessed using Begg’s rank correlation test and Egger’s regression test, alongside visual inspection of funnel plot symmetry.

To validate and contrast our cohort against pooled international data, the PSA response proportion observed in our cohort was compared descriptively with the pooled quantitative estimate, and a forest plot was generated to visually display the two estimates side-by-side. Sensitivity analyses were performed to assess the robustness of the pooled estimate, excluding, in turn, (i) the study contributing disproportionately to between-study heterogeneity [14], (ii) the study using a structurally distinct PSMA ligand [17], (iii) the studies that reported PSA response as a decline of more than 50% rather than ≥50% [2,12] and (iv) a leave-one-out analysis, in which each study was excluded in turn.

All analyses were performed using STATA software, version 18 (StataCorp LLC, College Station, TX, USA).

### 4.5. Statistics

No formal sample size calculation was made for this retrospective observational study. PSA levels and other laboratory markers following [^225^Ac]Ac-PSMA-617 TAT were compared with baseline using a repeated-measures one-way analysis of variance (ANOVA). Kaplan–Meier survival curves were generated for both PFS and OS. Each biomarker was analyzed individually to determine its potential prognostic significance. Threshold levels were established based on the median values obtained after two cycles of therapy. Statistical significance was determined using *p*-values, with a threshold set at *p* < 0.05. Univariate survival analyses were performed by estimating Kaplan–Meier curves for each biomarker dichotomized at the median value after two cycles of therapy and comparing the two strata using the unstratified log-rank test. The statistical analyses were performed using GraphPad Prism 8.0.1 (GraphPad Software, Boston, MA, USA).

To further evaluate the individual prognostic value of PSA, PSMA PET-derived TTV, and routine blood biomarkers (hemoglobin, platelets, creatinine, eGFR, ALP, and LDH) on OS, a complementary univariate Cox proportional hazards regression was performed for each biomarker separately. While the primary Kaplan–Meier/log-rank analysis (Table 3) dichotomized each biomarker at its post-cycle-2 median value to compare two patient strata, the Cox regression treated each biomarker, allowing the full range of biomarker values, rather than only a binary above/below-median split, to inform the estimated association with OS. These two approaches are complementary rather than redundant: the log-rank method offers a clinically interpretable threshold-based comparison, while Cox regression tests whether the association holds across the biomarker’s full distribution and is less sensitive to the specific cut-point chosen. Performing both provides a more robust assessment of whether each biomarker’s prognostic association is a stable signal or an artifact of the chosen median split.

Given our cohort size and limited number of events (6 deaths), a multivariable Cox model incorporating multiple covariates simultaneously was not statistically stable and was therefore not pursued, consistent with established guidance that Cox regression requires approximately 10 observed events per covariate for reliable estimation [41].

Each biomarker was standardized (converted to units of one standard deviation, SD) prior to model fitting, allowing hazard ratios (HR) across biomarkers of different physiological scales to be directly compared. Given the limited number of events, Firth-penalized Cox regression was adopted a priori as the primary estimation method for each biomarker to reduce small-sample bias [42]. To quantify the uncertainty of each HR estimate given the small sample, a non-parametric bootstrap procedure was performed: 2000 resamples of the cohort were drawn with replacement, and the Firth-penalized Cox model was refit on each resample to generate an empirical 95% CI for the HR; this bootstrapped interval, rather than the model-based profile-likelihood interval, is reported as the primary measure of uncertainty, as the latter was found to be numerically unstable for biomarkers with strong effects at this event count. Standard (unpenalized) Cox regression was additionally performed for each biomarker as a comparator and is reported in Supplementary Table S1. All statistical analyses were performed in R (v4.6.1; packages survival, coxphf, boot).

## 5. Conclusions

This study regards TAT with [^225^Ac]Ac-PSMA-617 as a treatment option for mCRPC patients who had developed resistance to [^177^Lu]Lu-PSMA-617 therapy; however, the findings should be interpreted cautiously given the small sample size (n = 12), which limits statistical power and the generalizability of the conclusions. [^68^Ga]Ga-PSMA-11 PET imaging parameters and blood biomarkers may show useful exploratory prognostic associations with treatment efficacy and clinical outcomes in mCRPC patients receiving [^225^Ac]Ac-PSMA-617 TAT. Although PSA level monitoring is a widely used approach to evaluate treatment response, incorporating TTV measurements and blood biomarkers may provide additional insights into therapeutic effectiveness. While our pilot cohort could not demonstrate an independent prognostic value of imaging-based parameters such as TTV for OS, the concordant behavior of TTV and PSA observed in a subset of patients, together with prior evidence supporting the prognostic relevance of PSMA PET-derived tumor volume in larger radioligand therapy cohorts, suggests that integrating blood biomarkers with PSA and imaging parameters may offer enhanced prognostic insight. This hypothesis warrants confirmation in larger, prospective studies.

In addition, findings from our patient population suggested that a ≥50% decrease in PSA levels after TAT was associated with favorable treatment outcomes in subsequent cycles, indicating a potential exploratory prognostic association.

Among pre-therapeutic and on-treatment blood biomarkers evaluated, PSA and ALP showed the most consistent association with overall survival, with concordant findings across both median-dichotomized and continuous statistical approaches. Hemoglobin, platelet count, and creatinine showed significant associations with OS only in the dichotomized analysis, and their prognostic value should therefore be considered preliminary, warranting confirmation in larger cohorts before clinical application.

The tumor sink effect could be considered in subsequent [^225^Ac]Ac-PSMA-617 therapy cycles, as tumor burden reduction may increase salivary gland uptake, potentially worsening toxicity, and optimizing therapy with semi-quantitative PET analysis can balance efficacy and safety. Post-treatment dosimetry and the estimation of absorbed doses for organs at risk following TAT present a novel challenge due to the suboptimal quality of post-treatment [^225^Ac]Ac-PSMA-617 SPECT and planar images. Given this limitation, pre-treatment [^68^Ga]Ga-PSMA-11 PET/CT imaging may hold promise as a practical predictor of absorbed dose for organs at risk, offering a route to dosimetry-informed dose planning that circumvents the technical constraints of post-treatment SPECT; formally establishing this relationship in a dedicated dosimetry cohort represents a valuable direction for future research.

## Figures and Tables

**Figure 1:**
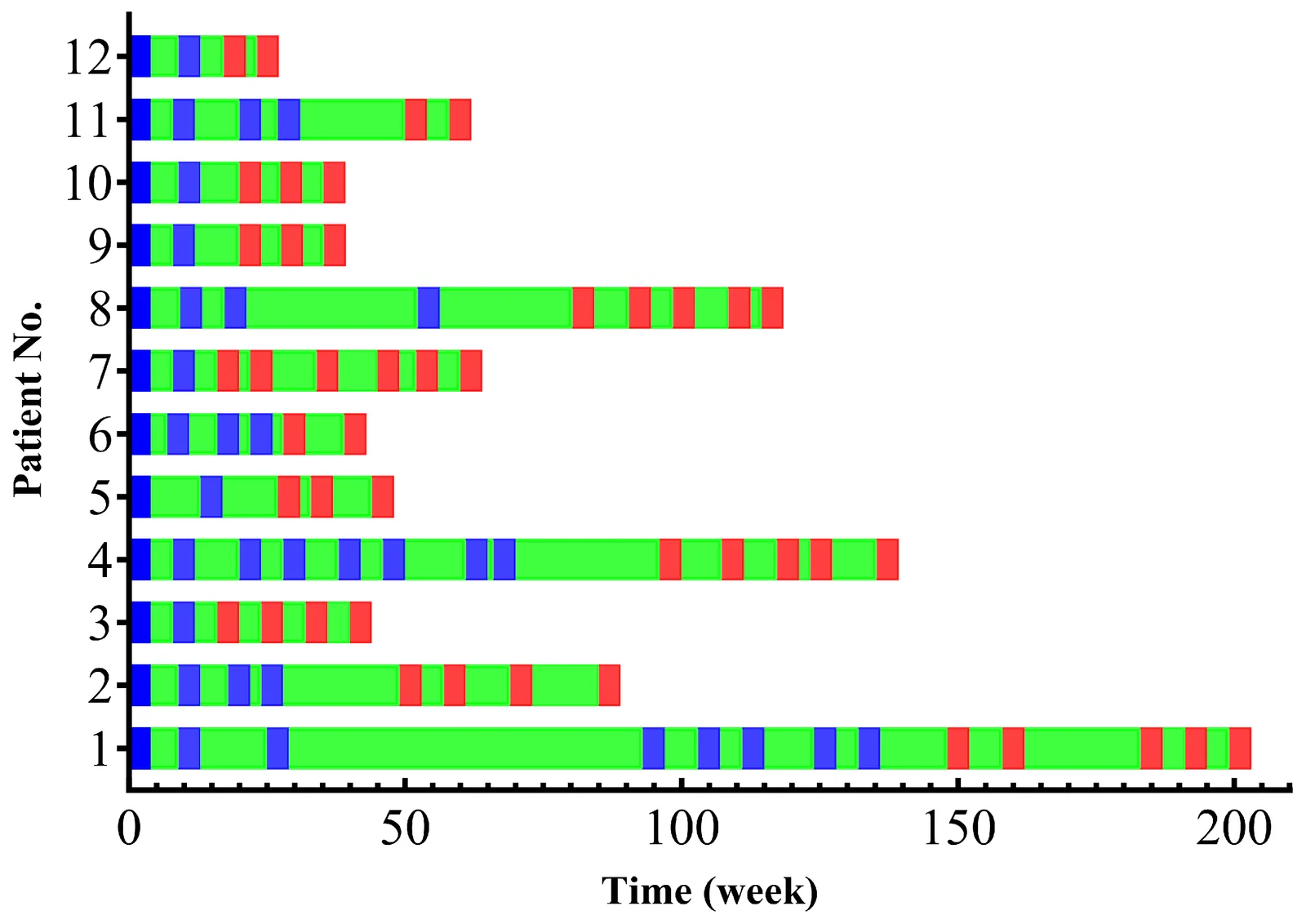
Swimmer plot illustrates the treatment timeline for each mCRPC patient, including administration time of [^177^Lu]Lu-PSMA-617 (blue), [^225^Ac]Ac-PSMA-617 (red), and pauses between therapies (green).

**Figure 2:**
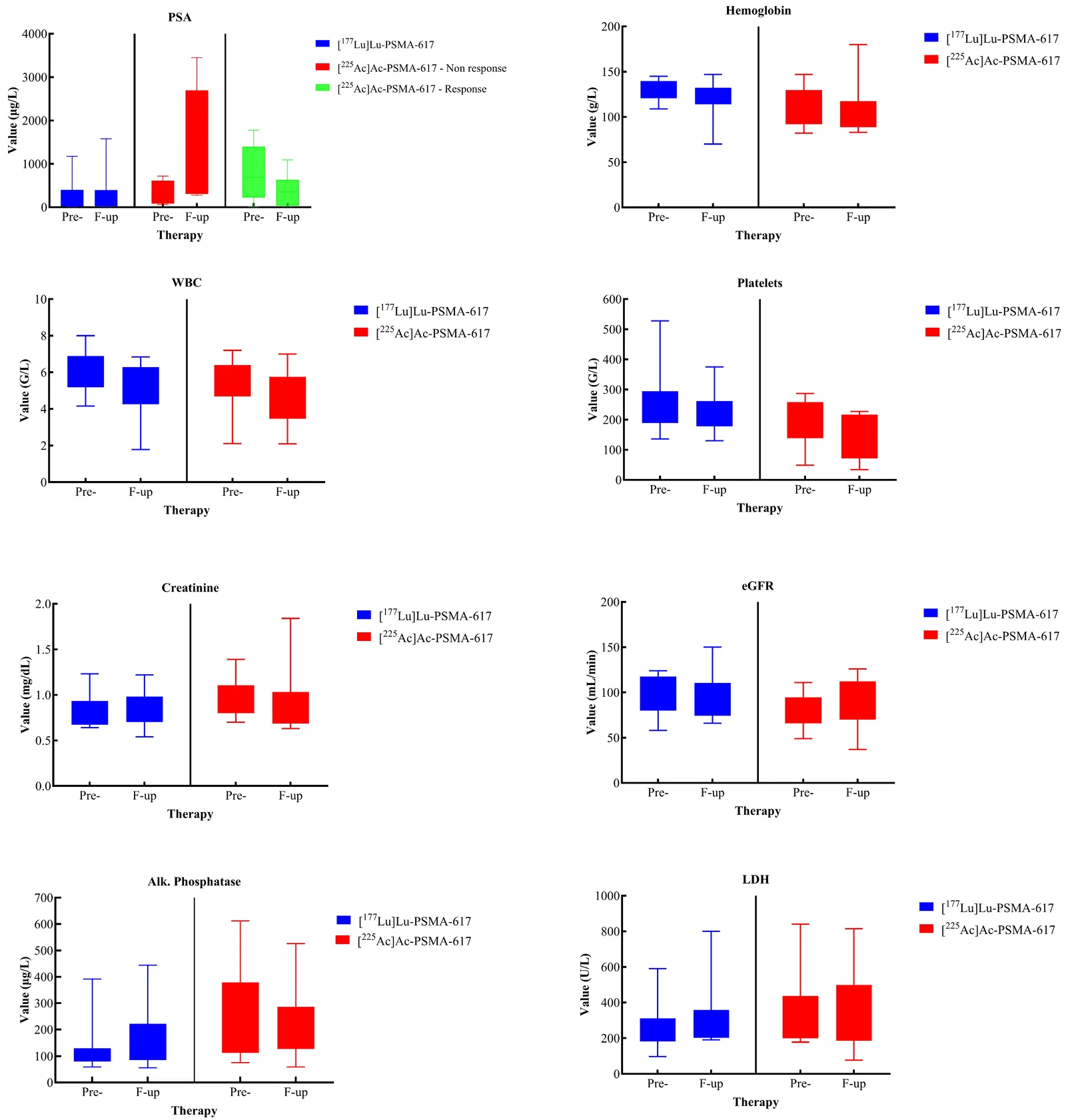
Box-and-whisker plots of hematologic, biochemical, renal, and PSA parameters before and after therapy. Panels show pre-therapy (“Pre-”) and last follow-up (“F-up”) values for each biomarker under [^177^Lu]Lu-PSMA-617 (blue) and [^225^Ac]Ac-PSMA-617 (red). PSA values are additionally stratified into non-responders (red) and responders (green). Each box represents the median, interquartile range, and full value range. Hemoglobin, WBC, platelets, creatinine, eGFR, alkaline phosphatase, and LDH showed no statistically significant changes between pre-therapy and follow-up for either radioligand therapy. PSA decreased in the responder group after [^225^Ac]Ac-PSMA-617 and increased in non-responders.

**Figure 3:**
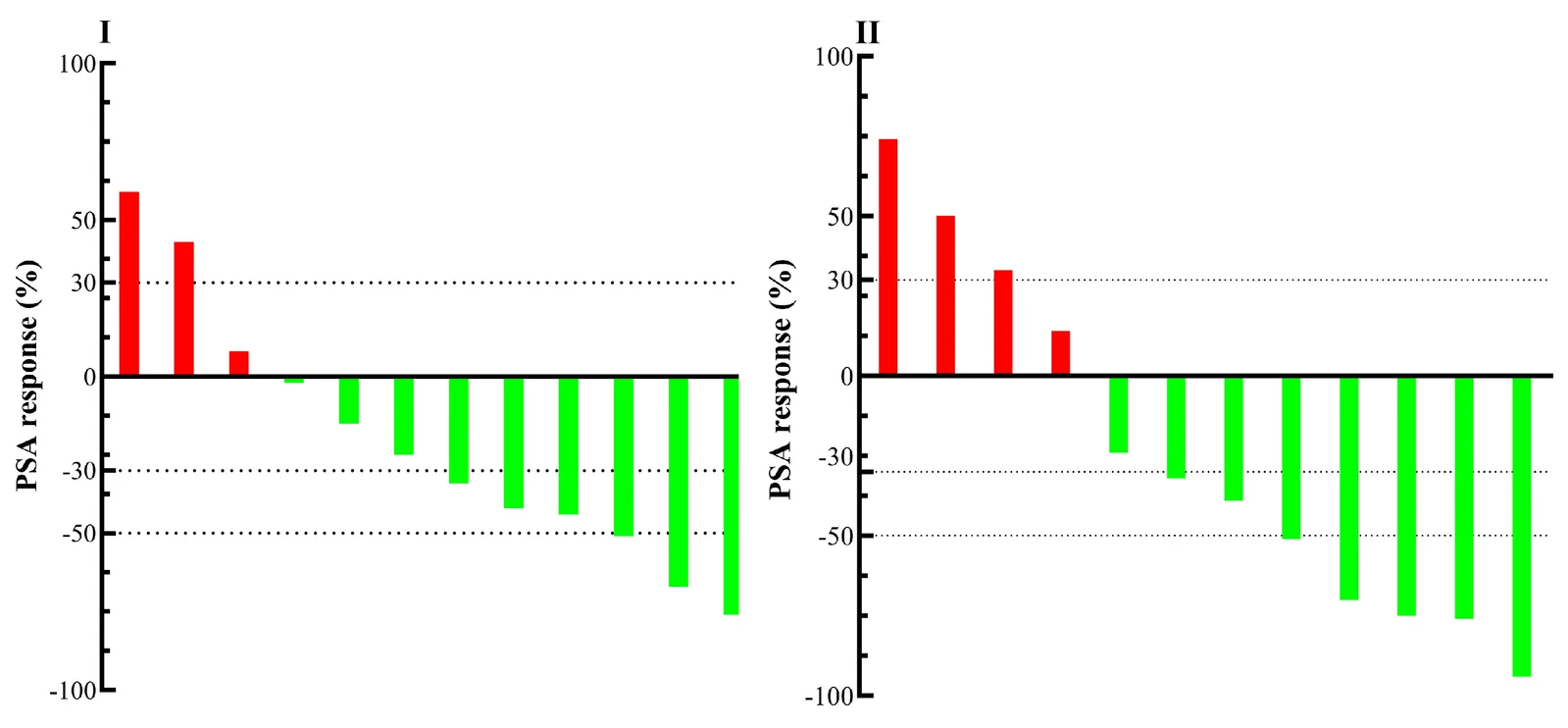
PSA-based biochemical response to [^225^Ac]Ac-PSMA-617 TAT. (**I**) Waterfall plot showing individual percentage changes in PSA levels after the first cycle of [^225^Ac]Ac-PSMA-617 and (**II**) PSA changes at the most recent follow-up. Patients with PSA decline are shown in green; those with PSA increase are shown in red. After the first cycle, 75% (9/12) of patients showed PSA decline, with 50% (6/12) achieving ≥30% and 25% (3/12) achieving ≥50% reduction. At follow-up, 67% (8/12) had a PSA decline, with 58% (7/12) showing ≥30% and 42% (5/12) showing ≥50% reduction. All 6 patients with an initial ≥30% decline maintained a ≥30% decline at follow-up; one additional patient reached ≥30% during follow-up.

**Figure 4:**
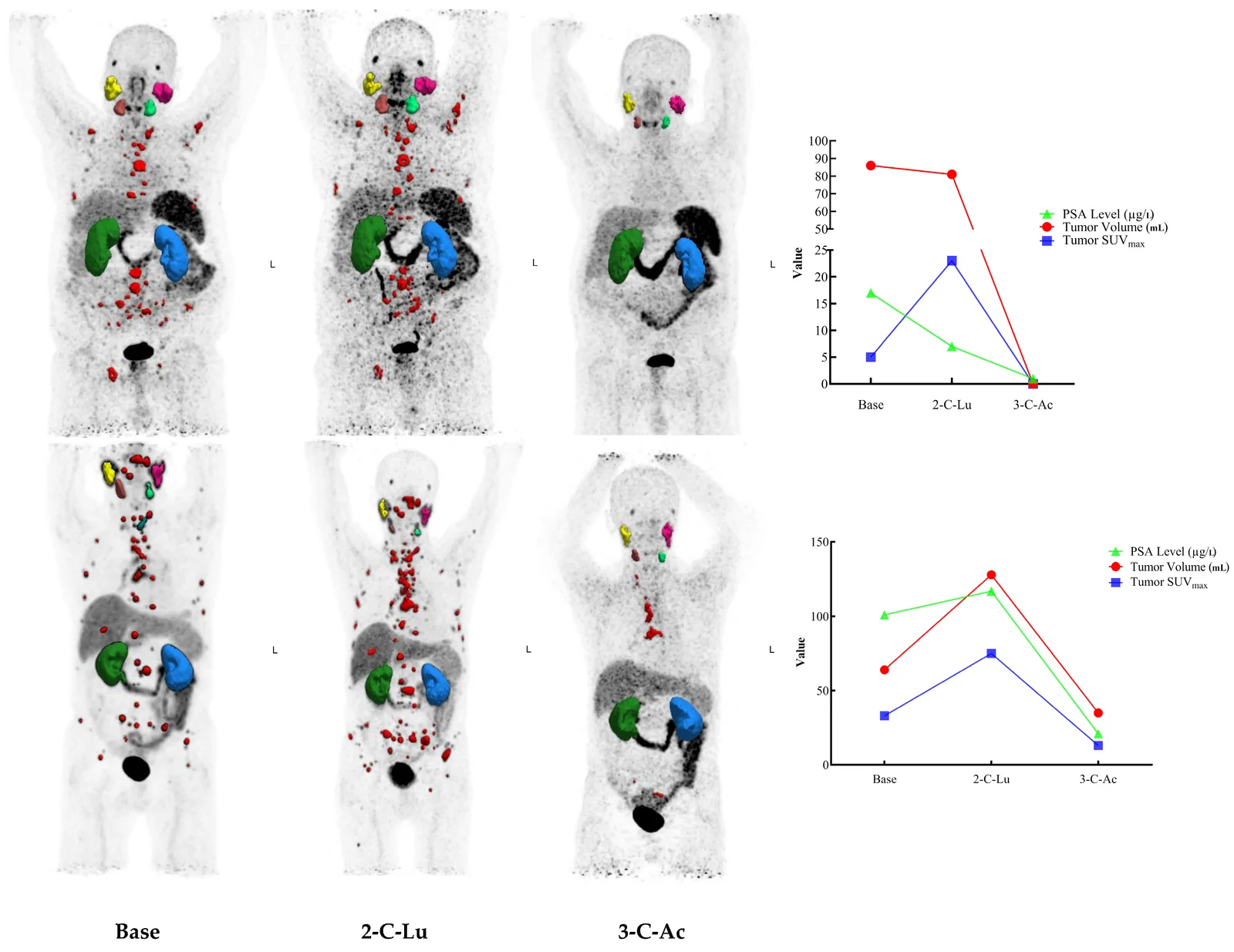
Molecular imaging and PSA response in two patients responding to TAT. [^68^Ga]Ga-PSMA-11 PET/CT images and schematic plots for two patients with mCRPC refractory to [^177^Lu]Lu-PSMA-617 therapy. (Base) Baseline scan; (2-C-Lu) after two cycles of [^177^Lu]Lu-PSMA-617; (3-C-Ac) after three cycles of [^225^Ac]Ac-PSMA-617. Graphs depict PSA level (µg/L, green), total tumor volume (mL, red), and tumor SUV_max_ (g/mL, blue). Both patients demonstrated near-complete molecular and biochemical responses, with PSA declining to undetectable levels and marked TTV reduction. There is no correlation between colors of graphs and volumes of interest in molecular PET images. In the PET images, red represents the tumor, blue the left kidney, dark green the right kidney, pink the left parotid gland, yellow the right parotid gland, light green the left salivary gland, and dark pink the right salivary gland. “L” indicates the anatomical left side.

**Figure 5:**
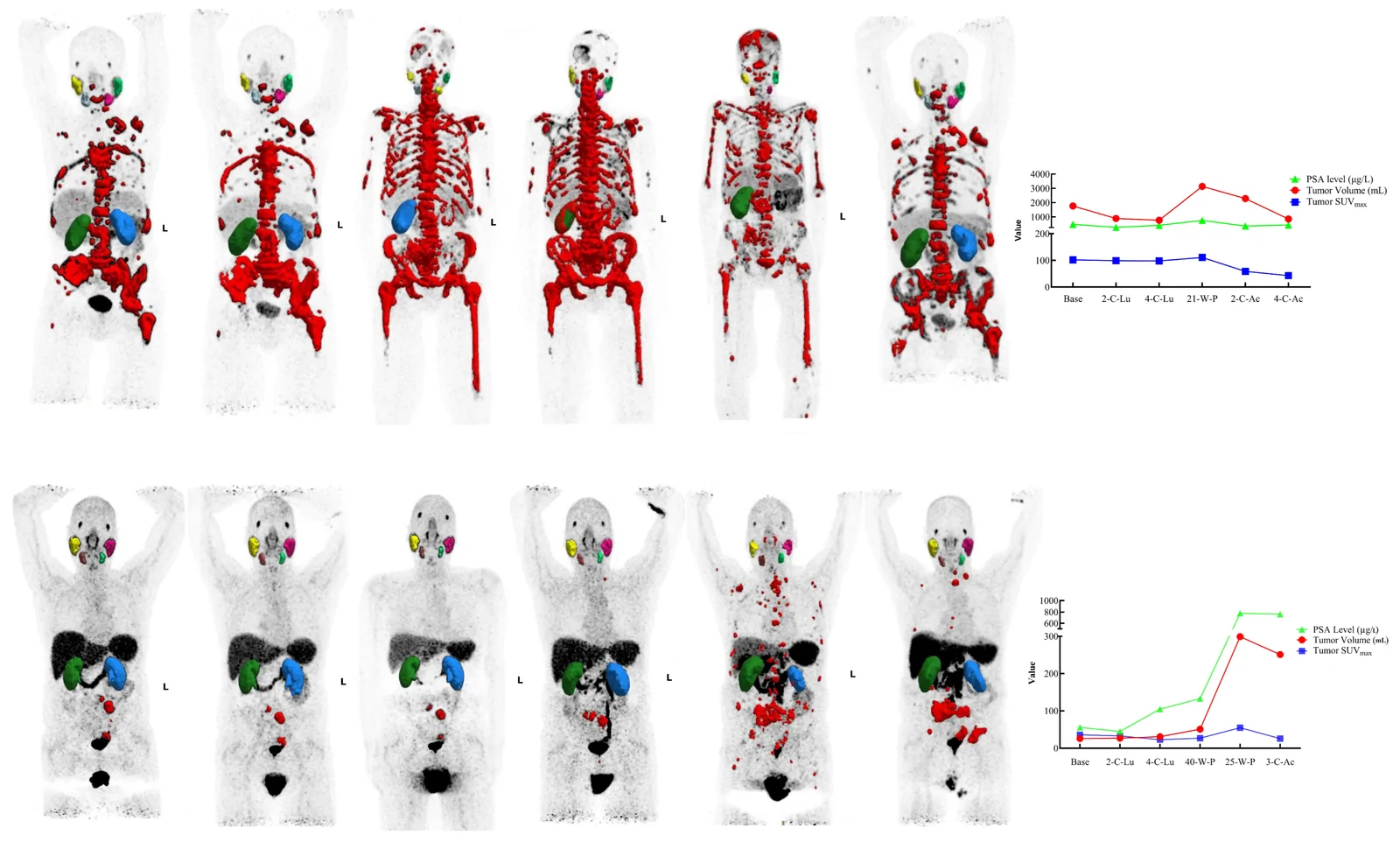
Disease stabilization after TAT in two patients with prior progression. [^68^Ga]Ga-PSMA-11 PET/CT images are shown for two patients with mCRPC who had progressed after [^177^Lu]Lu-PSMA-617 and underwent a long treatment pause. The top and bottom rows correspond to patient no. 2 and patient no. 8, respectively. (Base) baseline; (2-C-Lu) after two cycles of [^177^Lu]Lu-PSMA-617; (4-C-Lu) after four cycles of [^177^Lu]Lu-PSMA-617; (21-W-P) 21 weeks post-treatment pause; (40-W-P) 40 weeks post-treatment pause (2-C-Ac) after two cycles of [^225^Ac]Ac-PSMA-617; (25-W-P) 25 weeks post-treatment pause; (4-C-Ac) after four cycles of [^225^Ac]Ac-PSMA-617; (3-C-Ac) after three cycles of [^225^Ac]Ac-PSMA-617. Graphs show PSA level (µg/L, green), TTV (mL, red), and SUV_max_ (g/mL, blue). In these two illustrative cases, changes in PSA level appeared to parallel changes in TTV; TTV trends in these two cases were consistent with the SUV_peak_-based molecular response classification applied in this study (see Section 4.3), although this qualitative, case-level observation was not tested with a formal correlation analysis across the cohort. Both patients exhibited halting of disease progression and partial molecular response following TAT. Notably, the first patient (top row) presented with bone metastases. There is no correlation between colors of graphs and volumes of interest in molecular imaging PET. In the PET images, red represents the tumor, blue the left kidney, dark green the right kidney, pink the left parotid gland, yellow the right parotid gland, light green the left salivary gland, and dark pink the right salivary gland. “L” indicates the anatomical left side.

**Figure 6:**
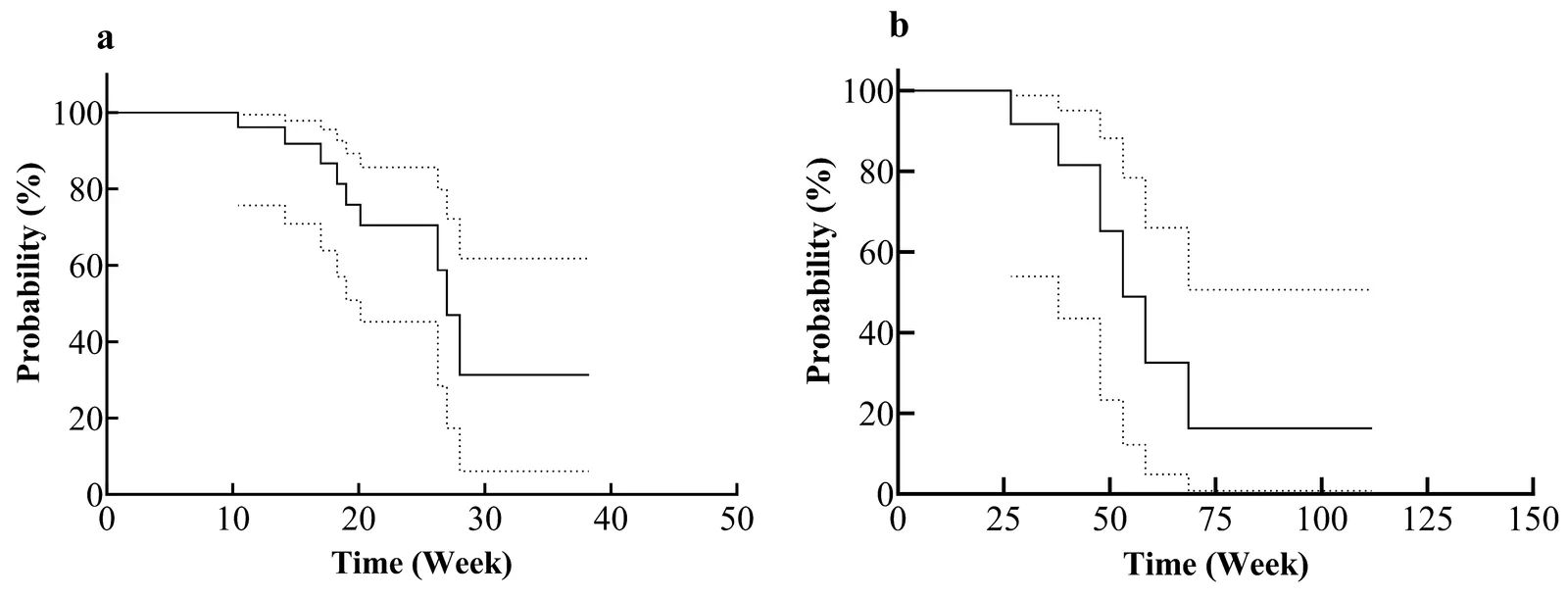
Kaplan–Meier survival analysis after [^225^Ac]Ac-PSMA-617 therapy. Kaplan–Meier plots showing (**a**) progression-free survival (PFS) and (**b**) overall survival (OS) in mCRPC patients treated with [^225^Ac]Ac-PSMA-617 TAT. Median PFS was 27 weeks (95% CI: 23–31 weeks). Median OS was 53 weeks (95% CI: 36–70 weeks). Survival probabilities were: 31% for PFS >30 weeks, 50% for OS >50 weeks, and 16% for OS >100 weeks. The dark lines represent Kaplan–Meier survival curves, while lighter lines indicate the upper and lower bounds of the 95% confidence intervals.

**Figure 7:**
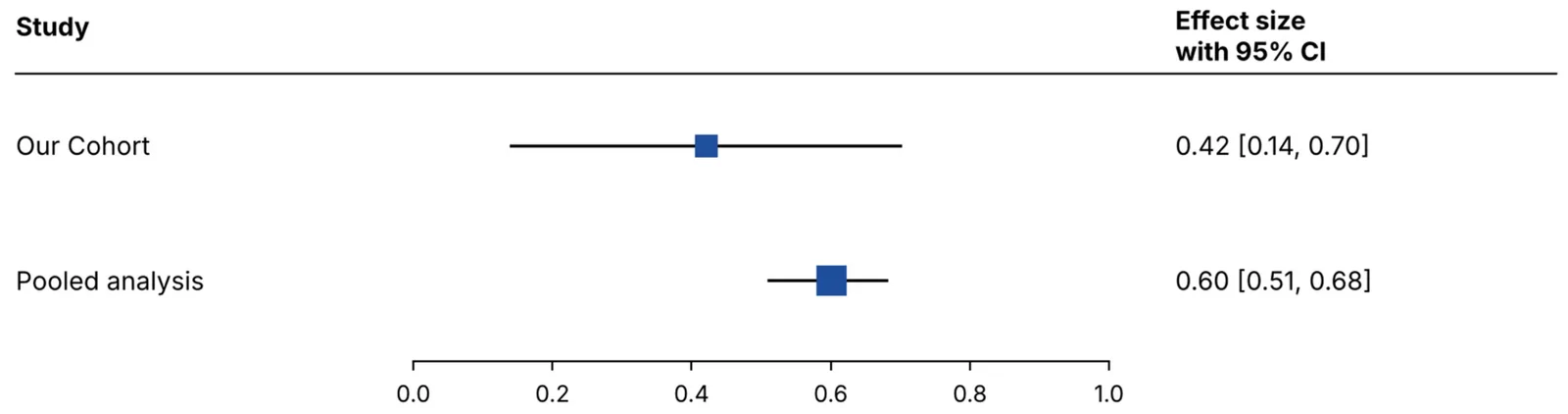
Contextualizing our cohort outcomes against pooled quantitative analysis benchmarks. Forest plot comparing the retrospective cohort (n = 12) outcomes to pooled estimates from n = 745 patients across ten studies.

**Figure 8:**
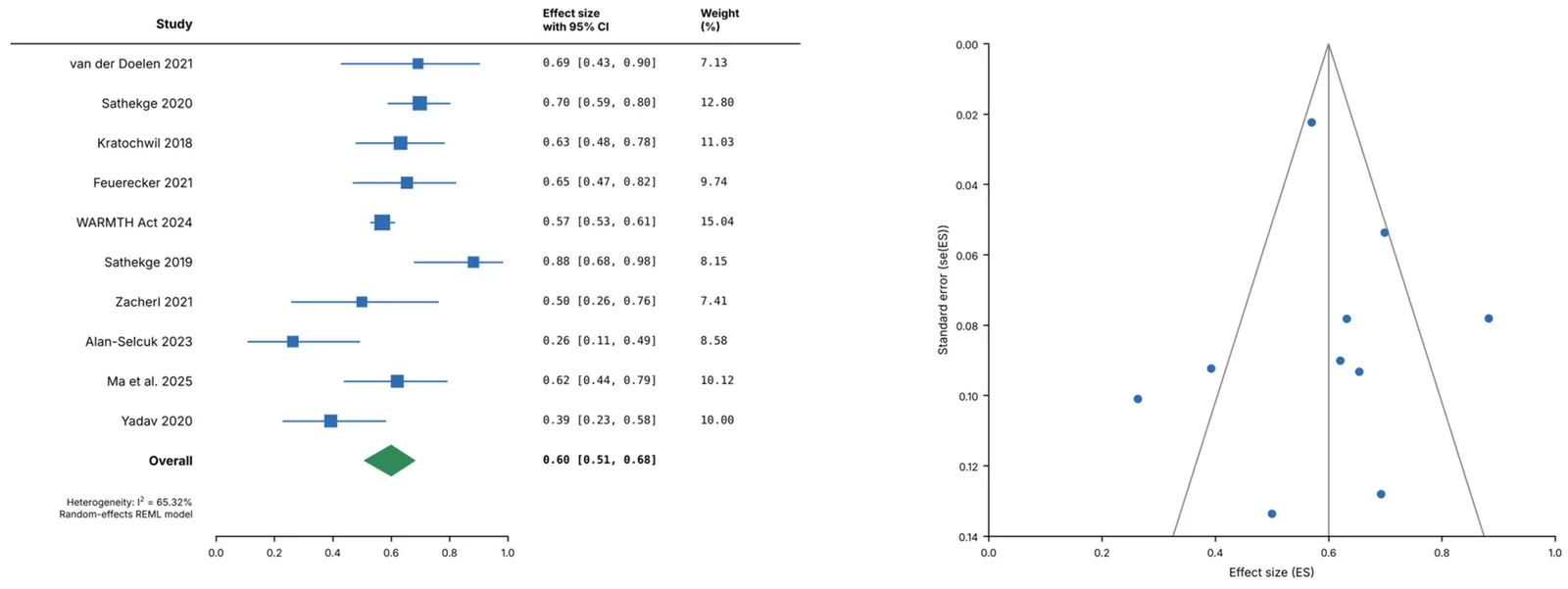
The pooled results (**left**) present the PSA response rate for patients achieving a ≥50% decline (ES = 0.60, 95% CI: 0.51–0.68) and includes a funnel plot (right) assessing publication bias. Begg’s test (τ = −0.20, *p* = 0.48) and Egger’s test (bias = 0.23, SE = 1.15, *p* = 0.85) did not indicate significant funnel plot asymmetry; however, given the limited number of included studies (n = 10) [2,7,10,12,13,14,15,16,17,25], these results should be interpreted as inconclusive rather than definitive evidence against publication bias. I^2^ = 65.32%, suggesting substantial heterogeneity, consistent with the sensitivity analysis presented above. X-axis (ES) shows the effect size from each study and Y-axis (se(ES)) represents the standard error of each study’s effect size.

**Table 1:** Clinical characteristics and prior treatments of mCRPC patients prior to [^225^Ac]Ac-PSMA-617 targeted alpha therapy, including age, barthel index, performance status, previous therapies, and metastatic sites at time of TAT.

Characteristic	Data
**Demographics** **Age (years)**	
Median (min–max)	72 (63–81)
**Barthel index, median (range)**	
Before [^177^Lu]Lu-PSMA-617 RLT	100 (95–100)
Before [^225^Ac]Ac-PSMA-617 TAT	100 (95–100)
**Previous Therapies, n (%)**	
**Radical prostatectomy**	8/12 (67%)
**Local Radiotherapy**	5/12 (42%)
**Hormone Therapy**	
Enzalutamide	10/12 (83%)
Abiraterone	9/12 (75%)
Trenantone	9/12 (75%)
Bicalutamide	2/12 (17%)
**Chemotherapy**	
Docetaxel	8/12 (67%)
Cabazitaxel	5/12 (42%)
Contraindication for chemotherapy (inclusive refusal by patient)	4/12 (33%)
**Radioligand Therapy**	
[^223^Ra]RaCl_2_	1/12 (8%)
[^177^Lu]Lu-PSMA-617: Number of cycles: median (min–max)	12/12 (100%) 4 (2–8)
**Targeted Alpha Therapy**	
[^225^Ac]Ac-PSMA-617: Number of cycles: median (min–max)	4 (2–6)
Individual activity (MBq): median (min–max)	8.0 (5.0–11.0)
**Metastatic sites at time of TAT, n (%)**	
Bone	12/12 (100%)
Regional lymph node	6/12 (50%)
Liver	2/12 (17%)
Lung	1/12 (8%)

**Table 2:** Baseline serum biomarkers and hematologic parameters in mCRPC patients prior to [^177^Lu]Lu-PSMA-617 and [^225^Ac]Ac-PSMA-617 therapy.

Laboratory Blood Test	[^177^Lu]Lu-PSMA-617 Initiation: Median (Min–Max)	[^225^Ac]Ac-PSMA-617 Initiation: Median (Min–Max)
PSA (μg/L)	87.4 (9.1–1177)	417.5 (7.1–1775)
Alk. Phosphatase (U/L)	96.5 (59–391)	194.5 (75–612)
Platelets (G/L)	224.0 (136–528)	229.5 (49–287)
Creatinine (mg/dL)	0.8 (0.6–1.2)	0.9 (0.7–1.4)
WBC (G/L)	6.2 (4.2–8)	5.8 (2.1–7.2)
eGFR (mL/min)	102.0 (58–124)	81.5 (49–111)
LDH (U/L)	204.0 (98–590)	296.5 (178–840)
Hemoglobin (g/L)	130.0 (109–145)	118.5 (82–147)

**Table 3:** Univariate analysis of PSA, hematologic biomarkers and tumor biomarkers after 2 cycles of [^225^Ac]Ac-PSMA-617, association with overall survival in mCRPC.

Variable	Threshold	OS, Median (Weeks)	95% CI	*p* Value	Significance
**PSA**	≥319 µg/L	43.5	27–59	0.002	**
	<319 µg/L	50	38–112	-	-
**Hemoglobin**	≥108 g/L	56.5	31–112	-	-
	<108 g/L	42.5	27–50	<0.001	***
**Platelets**	≥180 G/L	49	27–112	-	-
	<180 G/L	42.5	38–69	<0.001	***
**Creatinine**	≥0.84 mg/dL	50	46–112	-	-
	<0.84 mg/dL	38	27–54	0.003	**
**eGFR**	≥89 mL/min	38.5	27–69	0.084	ns
	<89 mL/min	49	46–112	-	-
**ALP**	≥163 U/L	42.5	27–59	0.012	*
	<163 U/L	52	31–112	-	-
**LDH**	≥268 U/L	42	27–54	0.014	*
	<268 U/L	54.5	39–112	-	-

Table threshold level has been determined based on the median of results available for 12 patients at the post-cycle-2 assessment (* [*p* ≤ 0.05]; ** [*p* ≤ 0.01]; *** [*p* ≤ 0.001]; ns: not statistically significant).

**Table 4:** Univariate Cox regression of overall survival by post-cycle-2 biomarker values (Firth-penalized HR per 1-SD increase, bootstrapped 95% CI, 2000 resamples).

Biomarker	N	Events	HR (Firth, per-SD)	Bootstrap 95% CI
PSA	12	6	2.18	1.06–23.57
ALP	12	6	3.73	1.07–27.72
Hemoglobin	12	6	0.46	0.11–1.04
Creatinine	12	6	0.55	0.10–1.63
eGFR	12	6	2.04	0.66–7.70
LDH	12	6	1.94	0.78–12.23
Platelets	12	6	0.88	0.48–6.49
WBC	12	6	0.88	0.45–2.44
TTV *	9	6	1.16	0.47–1772.90

* TTV was entered into the survival model as the absolute post-cycle-2 tumor volume (mL), rather than as a percentage change from baseline. Given the substantial inter-patient variability in absolute tumor burden and the small cohort size, this estimate, like all associations reported in this table, should be interpreted as exploratory and hypothesis-generating rather than as a definitive prognostic threshold.

**Table 5:** Summary of current and emerging strategies for the prevention and treatment of TAT-induced xerostomia, categorized by mechanism, stage of use, and evidence maturity [26].

Strategy	Mechanism	Stage of Use	Evidence/Effectiveness	Readiness Level
**External Cooling (Ice Packs)**	Vasoconstriction to reduce uptake	*Used in our cohort*	No significant reduction in gland SUV	Widely accessible but limited efficacy
**Oral Vitamin C (Ascorbic Acid)**	Parasympathetic stimulation to increase clearance	Investigational	26–52% decrease in gland SUV_mean_	Low-cost, promising data
**Botulinum Toxin A Injection**	Chemical denervation (temporary atrophy)	Investigational	~64% SUV reduction; saliva preservation in TAT patients	Clinically feasible, promising
**Monosodium Glutamate (MSG)**	Competitive inhibition at PSMA binding sites	Investigational	24–35% SUV_max_ reduction in glands	Early clinical trial phase
**Stem Cell Therapy**	Regeneration of gland tissue	In trials (Phase 1/2)	33–50% increase in saliva flow	High potential, not yet approved
**Pilocarpine/Cevimeline**	Muscarinic stimulation of saliva	Symptom palliation	Significant dry mouth relief in head/neck cancer trials	Routinely used in oncology practice
**ALTENS (Electrical Nerve Stimulation)**	Neuromodulation	Symptom palliation	Comparable to pilocarpine, fewer side effects	Available in select centers

**Table 6:** Comparison of Key Outcome Measures in Clinical Studies Using ^225^Ac-Labeled PSMA TAT ([^225^Ac]Ac-PSMA-617 and [^225^Ac]Ac-PSMA-I&T) for mCRPC.

Study (Authors)	Patient (#)	Injected Activity	Median PFS (Months)	Median OS (Months)	PSA Response (≥50%), n/N ^†^	Xerostomia (% of Patients)	Hematologic Toxicities (% of Patients)	Renal Toxicities (% of Patients)
Kratochwil et al. [11]	14	50–200 kBq/kg	None reported	None reported	^‡^ NA	None reported	Not specified	None reported
Kratochwil et al. [12]	40	100 kBq/kg	9	None reported	^¶^ 63%-24/38	None reported	None reported	None reported
Sathekge et al. [16]	17	4–13 MBq (escalation)	None reported	12	^§^ 88%-15/17	100% (Grade 1/2)	6% (Grade 3 anemia)	6% (Grade 4)
Yadav et al. [2]	28	100 kBq/kg	12	17	^¶^ 39%-11/28	29%	4% (Grade 3 anemia)	(Rate not reported) (Grade 1)
Sathekge et al. [10]	73	4–8 MBq (escalation)	15.2	18	70%-51/73	85%	Anemia in 37%	7% (Grade 3 or 4)
van der Doelen et al. [7]	13	None reported	None reported	8.5	69%-9/13	100%	None reported	None reported
Feuerecker et al. [13]	26	Median 9 MBq per cycle	3.5	7.7	65%-17/26	100% (Grade 1/2)	35% (anemia)	None reported
Zacherl et al. [17]	14	Median 7.8 MBq	None reported	None reported	50%-7/14	36% (Grade 1/2)	21% (Grade 3 anemia)	None reported
Alan-Selcuk et al. [14]	23	Mean 7.6 MBq	3.1	7.7	26%-5/19	100%	4% (Grade 3 anemia)	4% (Grade 3 nephrotoxicity)
Sathekge et al. [15]	488	Median 8 MBq	7.9	15.5	57%-278/488	68%	24% (13% Grade ≥3 anemia, 4% leukopenia, 7% thrombocytopenia)	5% (renal toxicity)
Ma et al. [25]	29	Median 8 MBq	8	18	62%-18/29	79%	Grade 3 in 24.1%	Creatinine/eGFR decline (stat. sig.), no Grade ≥3

^†^ n denotes the number of patients achieving a ≥50% PSA decline, and N denotes the number of patients evaluable for PSA response in each study ([12]: 38 of 40 treated patients; [14]: 19 of 23 treated patients). ^‡^ One study reported a composite response rate of 82% [11] (9/11 evaluable patients with either objective radiologic response or PSA decline); as this is not a discrete ≥ 50% PSA-decline outcome, it is not presented in this column (see Results, Section 2.6). This study’s PSA-response outcome is therefore listed as “NA (not applicable)” above. ^§^ One study did not report a discrete ≥50% PSA-decline count [16]. It was derived from the PSA data in the publication: 14/17 patients had a PSA decline of ≥90%, and one additional patient had a best PSA decline of 74% (patient no. 3; 204.7 to 53.3 ng/mL), giving 15/17. The two remaining patients had a PSA rise or a decline of <50% (as reported in Figure 1b by [16]). The >50% reduction in lesion tracer avidity on PSMA PET/CT reported in that study (15/17 patients) is a separate imaging outcome and was not used here. ^¶^ Response thresholds and time points are given as reported by each study. PSA response was defined as a decline of ≥50% from baseline, except in [12] (among patients surviving at least 8 weeks) and [2] (at the end of follow-up), which reported a decline of more than 50%. Both were treated as PSA50 response under a uniform harmonization rule, and a sensitivity analysis excluding them is reported in Section 2.6.

## Data Availability

The patient-level data analyzed in this study (institutional cohort) are not publicly available due to privacy and ethical restrictions, as they contain sensitive clinical information from patients treated at the University Hospital Marburg. De-identified data may be made available upon reasonable request from the corresponding author, subject to approval by the responsible ethics committee and applicable data protection regulations. The data used for the pooled analysis were extracted from previously published studies cited in the manuscript (Table 6).

## Supplementary Materials

The following supporting information can be downloaded at: https://www.mdpi.com/article/10.3390/ijms27209330/s1.

## Abbreviations

The following abbreviations are used in this manuscript:

^68^Ga	Gallium-68
^177^Lu	Lutetium-177
^223^Ra	Radium-223
^225^Ac	Actinium-225
ADT	Androgen Deprivation Therapy
ALP	Alkaline Phosphatase
CI	Confidence Interval
CTCAE	Common Terminology Criteria for Adverse Events
eGFR	estimated Glomerular Filtration Rate
HR	hazard ratio
LDH	Lactate dehydrogenase
mCRPC	metastatic Castration-Resistant Prostate Cancer
OS	Overall Survival
PET/CT	Positron Emission Tomography/Computed Tomography
PFS	Progression-Free Survival
PRISMA	Preferred Reporting Items for Systematic Reviews and Meta-Analyses
PSA	Prostate-Specific Antigen
PSMA	Prostate-Specific Membrane Antigen
REML	Restricted Maximum Likelihood
RLT	Radioligand Therapy
SD	standard deviation
SGU	Salivary Gland Uptake
SPECT	Single-Photon Emission Computed Tomography
SUV	Standardized Uptake Value
TAT	Targeted Alpha Therapy
TTV	total tumor volume
WBC	White Blood Cell
