# Supplementary material for: Multimodal Evaluation of [225Ac]Ac-PSMA-617 Therapy in mCRPC: PET Based Total Tumor Volume, PSA Kinetics, and Blood Biomarkers
Source: Int J Mol Sci. 2026 Oct 20;27(20):9330. doi: 10.3390/ijms27209330 (PMC13705418; doi:10.3390/ijms27209330)
Supplement: Supplementary file 1 [file ijms-27-09330-s001.zip › ijms-4504967-supplementary.pdf]

International Journal of
Molecular Sciences

MDPI

Supplementary Materials

Multimodal Evaluation of [^{225}Ac]Ac-PSMA-617 Therapy in mCRPC: PET Based Total Tumor Volume, PSA Kinetics, and Blood Biomarkers

Ali Ebrahimifard, Shamim Bagheri, Qi Wang, Azimeh Hojjat Shamami, Pietro Di Fazio, Friederike Eilsberger, Laura Müller, Markus Luster, Damiano Librizzi and Behrooz Hooshyar Yousefi *

S1. Radiopharmaceutical preparation

Vipivotide tetraxetan (PSMA-617, 10µg/GBq) was added to 1 mL of 0.1M sodium ascorbate stock solution. The mixed solution was added into the vial containing [^{225}Ac]$AcCl_3$ (ITM Isotope Technologies, Garching, Germany), then was shaken gently. Then, the vial was heated at 90 ˚C for 25 minutes. After cooling to room temperature, the [^{225}Ac]Ac-PSMA-617 product was diluted with 5 mL of NaCl and filtered through a sterile filter.

Instant radio thin-layer chromatography (rTLC) was performed for quality control at room temperature by pipetting 5µL of the final product onto an iTLC-SA strip using Natrium-Citrate-buffer and an iTLC-SG strip using Ammonium acetate (MeOH-NH_4Ac) buffer. The radiochemical yield was determined using a TLC scanner Marita Star (Elysia Raytest) ≥ 95%, and the pH was 7–8. Additionally, bacterial endotoxin level was tested <10 EU/mL using the Endosafe nexgen-PTS (Charles River) before releasing for patient application.

S2. Univariate Cox regression analysis

Table S1. Comparison of standard and Firth-penalized univariate Cox regression estimates, with bootstrapped 95% confidence intervals, for PSA, PSMA PET-derived TTV, and routine blood biomarkers as predictors of overall survival (per 1-SD increase).

Biomarker	N	Events	HR (standard Cox)	95% CI (standard)	p (standard)	HR (Firth)	p (Firth)	HR (bootstrap, median)	Bootstrap 95% CI
PSA	12	6	2.36	0.86–6.47	0.095	2.18	0.081	2.19	1.06–23.57
ALP	12	6	4.24	1.21–14.81	0.024	3.73	0.012	3.42	1.07–27.72
Hemoglobin	12	6	0.41	0.14–1.24	0.114	0.46	0.104	0.47	0.11–1.04
Creatinine	12	6	0.49	0.14–1.80	0.285	0.55	0.348	0.52	0.10–1.63
eGFR	12	6	2.19	0.70–6.78	0.176	2.04	0.177	2.20	0.66–7.70
LDH	12	6	1.92	0.76–4.84	0.167	1.94	0.141	2.05	0.78–12.23
Platelets	12	6	0.85	0.39–1.84	0.673	0.88	0.722	0.97	0.48–6.49
WBC	12	6	0.85	0.37–1.93	0.692	0.88	0.741	0.88	0.45–2.44
TTV*	9	6	1.08	0.53–2.20	0.837	1.16	0.647	1.21	0.47–1772.90

*TTV was available for only 9/12 patients due to missing imaging data (n = 3); the extremely wide bootstrap interval reflects the reduced effective sample size and should not be interpreted as a stable estimate.

Model-based confidence intervals for the Firth-penalized model were numerically unstable for biomarkers with strong effects at this event count (profile-likelihood convergence issues at n = 12, 6 events) and are therefore not reported; the bootstrapped confidence

interval is used as the primary measure of uncertainty throughout the main text and this table, consistent with the Methods.

S3. Patient flow diagram for the current analysis

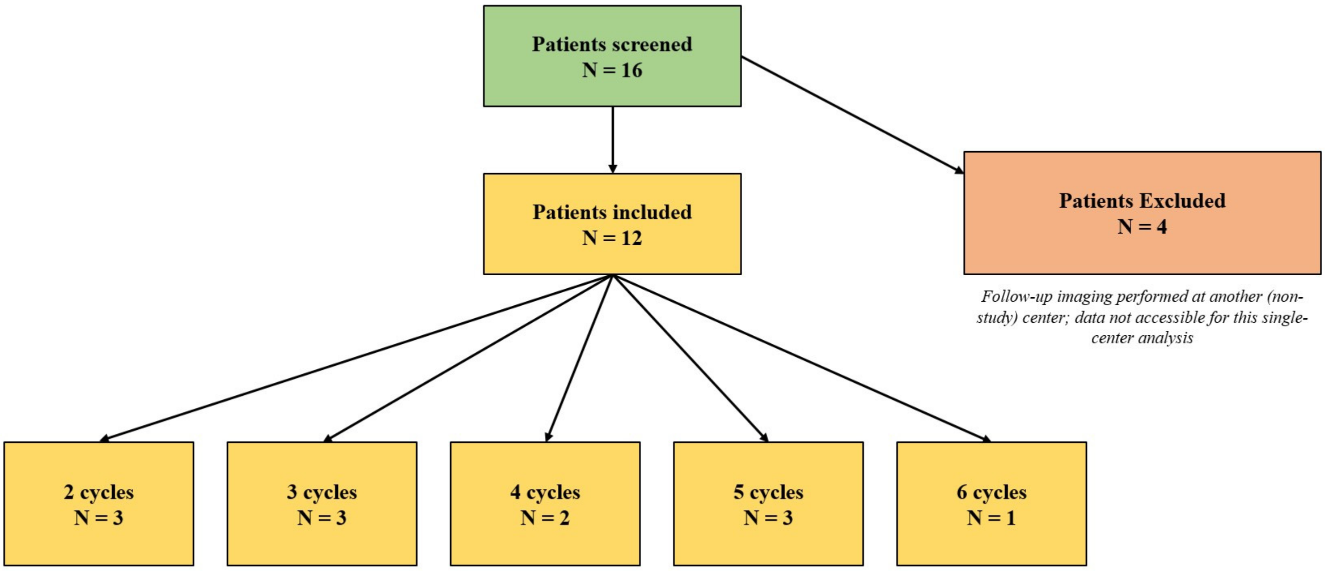

Figure S1. Of the 16 patients with mCRPC treated with [^{225}Ac]Ac-PSMA-617 tumor-targeted alpha therapy (TAT) after inadequate response to [^{177}Lu]Lu-PSMA-617 radioligand therapy (RLT), four patients were excluded because their follow-up molecular imaging was performed at another, non-study center and was therefore not accessible for this single-center analysis. The remaining 12 patients were included in the final analysis and received a total of 2–6 treatment cycles (2 cycles, n = 3; 3 cycles, n = 3; 4 cycles, n = 2; 5 cycles, n = 3; 6 cycles, n = 1).

S4. PRISMA flow diagram for the pooled quantitative analysis

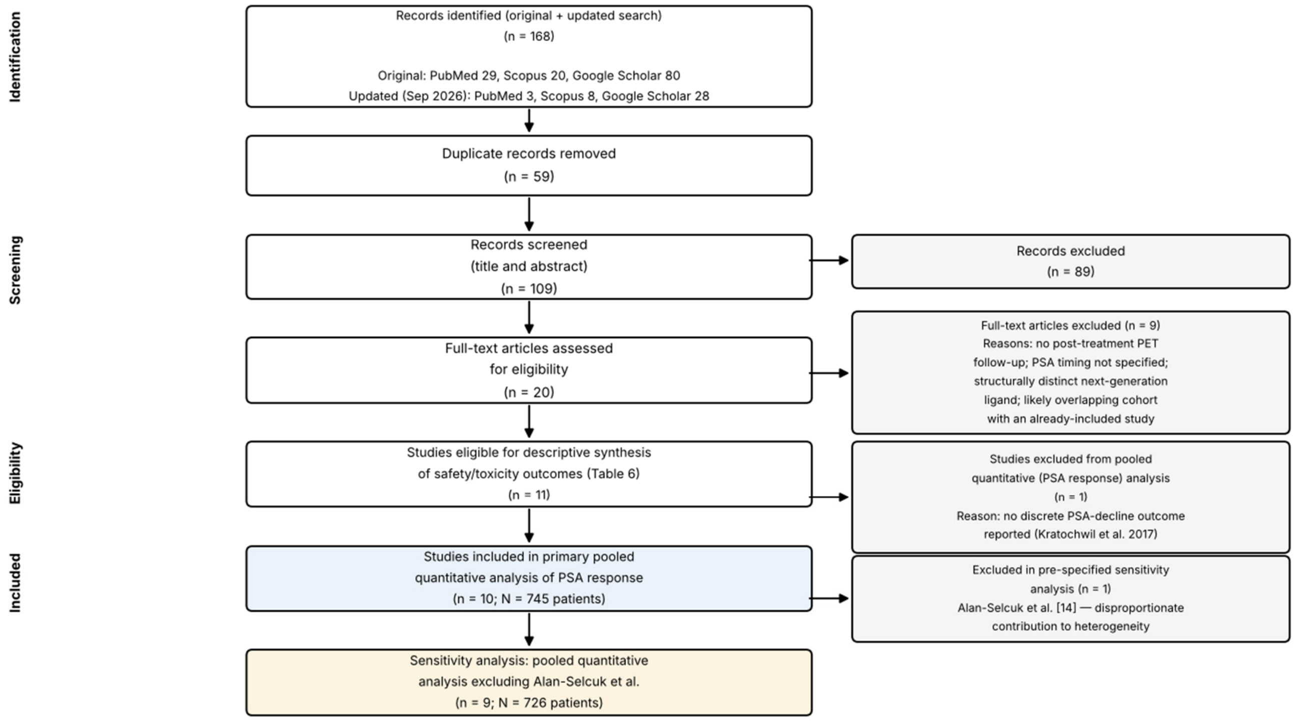

Figure S2. PRISMA-style flow diagram summarizing the literature search and study-selection process underlying the pooled quantitative analysis of PSA response in patients with mCRPC treated with [^{225}Ac]Ac-PSMA TAT. The full search strategy is reported in Methods (Section 4.4). The original search, conducted on 15 July 2026, identified 129 records through PubMed (n = 29), Scopus (n = 20), and Google Scholar (n = 80); an updated search using the identical strategy, conducted on 15 September 2026, identified a further 39 records through PubMed (n = 3), Scopus (n = 8), and Google Scholar (n = 28), for a combined total of 168 records. After removal of 59 duplicates, 109 records were screened by title and abstract, of which 89 were excluded. Seventeen full-text articles from the original search and 3 full-text articles from the updated search (20 in total) were assessed for eligibility; 9 were excluded because they lacked post-treatment PET imaging at follow-up, did not specify the timing of PSA measurement after therapy, reported on a structurally distinct next-generation PSMA ligand, or were judged likely to represent an overlapping patient cohort with an already-included study from the same institution, leaving 11 studies eligible for the descriptive synthesis of safety and toxicity outcomes (Table 6). Of these, 1 study (Kratochwil et al. 2017 [11]) did not report a discrete PSA-decline outcome and was therefore not eligible for the pooled quantitative analysis of PSA response, leaving 10 studies (N = 745 patients) included in the primary pooled quantitative analysis. A sensitivity analysis excluding Alan-Selcuk et al. [14], which contributed disproportionately to between-study heterogeneity, yielded 9 studies (N = 726 patients).

S5. Risk-of-bias assessment for the pooled quantitative analysis

Table S2. Risk-of-bias assessment of the 10 studies included in the pooled quantitative analysis of PSA response, using a Newcastle-Ottawa Scale (NOS) adapted for single-arm cohort studies. The Comparability domain of the standard NOS was not applicable, as none of the included studies had a concurrent comparator arm; scoring was therefore restricted to the Selection (max 3 stars) and Outcome (max 3 stars) domains, for a maximum total of 6 stars.

Study (Author, Year)	Selection (max 3)	Outcome (max 3)	Total (max 6)	Notes
Yadav et al., 2020 [2]	★★★	★★★	6/6	Prospective cohort; 3 of 31 consecutive patients (10%) excluded because of no follow-up after the first cycle, below the common 20% attrition threshold.
van der Doelen et al., 2021 [7]	★★★	★★★	6/6	Consecutive patients; full cohort (N=13) accounted for in the PSA-response denominator
Sathekge et al., 2020 [10]	★★★	★★★	6/6	Consecutive patients; full cohort (N=73) accounted for in the PSA-response denominator
Kratochwil et al., 2018 [12]	★★★	★★★	6/6	2/40 patients (5%) died before 8-week PSA evaluability; reason documented, below common 20% attrition threshold
Feuerecker et al., 2021 [13]	★★★	★★★	6/6	Consecutive patients; full cohort (N=26) accounted for in the PSA-response
Alan-Selcuk 2023 [14]	★★★	★★☆	5/6	PSA evaluable in 19/23 patients (4 not evaluable, reason not fully specified in the source publication); included in the primary pooled analysis, with its exclusion examined in a sensitivity analysis due to its disproportionate contribution to between-study heterogeneity
Sathekge et al. (WARMTH Act), 2024 [15]	★★★	★★★	6/6	Large multicentre cohort (N=488); largest and most representative study in the pooled analysis
Sathekge et al., 2019 [16]	★★★	★★★	6/6	Chemotherapy-naive population, in contrast to the other 9 studies, in which a substantial proportion of patients had received prior chemotherapy; noted as a potential source of clinical heterogeneity rather than a risk-of-bias concern
Zacherl et al., 2021 [17]	★★★	★★☆	5/6	4/18 patients (22%) excluded from analysis due to inaccessible outside medical records, exceeding the common 20% attrition threshold
Ma et al. 2025 [46]	★★★	★★★	6/6	Consecutive patients; full cohort (n = 29) accounted for in the PSA-response denominator; identified through the updated literature search

https://doi.org/10.3390/ijms27209330

Scoring criteria: Selection domain — representativeness of the cohort (consecutive/representative patients), secure ascertainment of exposure (documented radiopharmaceutical administration), and demonstration that the outcome was not present at baseline (documented progressive mCRPC prior to treatment). Outcome domain, objective assessment of outcome (PSA measured by standardized laboratory assay), adequacy of follow-up duration for the outcome to occur, and adequacy of cohort follow-up (denominator fully accounted for, with exclusions explained and below 20%, where applicable). Each star (★) indicates that a study fully met the corresponding methodological criterion within a domain; a half-mark (☆) indicates the criterion was only partially met. A higher total star count reflects a lower risk of bias.

https://doi.org/10.3390/ijms27209330
